# Locus coeruleus tonic upregulation increases selectivity to inconspicuous auditory information in autistic compared to non-autistic individuals: a combined pupillometry and electroencephalography study

**DOI:** 10.1186/s13229-025-00678-w

**Published:** 2025-08-21

**Authors:** Nico Bast, Jumana Ahmad, Luke Mason, Emily J. H. Jones, Magdalena Matyjek, Leonie Polzer, Christina Luckhardt, Anna Katharina Müller, Grainne M. McAlonan, Tobias Banaschewski, Sarah Baumeister, Eva Loth, Christine M. Freitag, The EU-AIMS LEAP group consists of, Nico Bast, Jumana Ahmad, Luke Mason, Emily J. H. Jones, Tobias Banaschewski, Sara Ambrosino, Bonnie Auyeung, Simon Baron-Cohen, Sarah Baumeister, Christian F. Beckmann, Sven Bölte, Thomas Bourgeron, Carsten Bours, Michael Brammer, Daniel Brandeis, Claudia Brogna, Yvette de Bruijn, Jan K. Buitelaar, Bhismadev Chakrabarti, Tony Charman, Ineke Cornelissen, Daisy Crawley, Flavio Dell’Acqua, Guillaume Dumas, Sarah Durston, Christine Ecker, Jessica Faulkner, Vincent Frouin, Pilar Garcés, David Goyard, Lindsay Ham, Hannah Hayward, Joerg Hipp, Rosemary Holt, Mark Johnson, Prantik Kundu, Meng-Chuan Lai, Xavier Liogier D’ardhuy, Michael V. Lombardo, Eva Loth, David J. Lythgoe, René Mandl, Andre Marquand, Maarten Mennes, Andreas Meyer-Lindenberg, Carolin Moessnang, Declan G. M. Murphy, Bethany Oakley, Laurence O’Dwyer, Marianne Oldehinkel, Bob Oranje, Gahan Pandina, Antonio M. Persico, Barbara Ruggeri, Amber Ruigrok, Jessica Sabet, Roberto Sacco, Antonia San José Cáceres, Emily Simonoff, Will Spooren, Julian Tillmann, Roberto Toro, Heike Tost, Jack Waldman, Steve C. R. Williams, Caroline Wooldridge, Marcel P. Zwiers

**Affiliations:** 1 Department of Child and Adolescent Psychiatry, Psychosomatics and Psychotherapy, Autism Research and Intervention Center of Excellence, University Hospital Frankfurt, Goethe-University, Deutschordenstraße 50, 60528 Frankfurt Am Main, Germany; 2 School of Human Sciences, University of Greenwich, London, UK; 3 Institute of Psychiatry, Psychology and Neuroscience, King’s College, London, London, UK; 4 Centre for Brain and Cognitive Development, Birkbeck College, University of London, Malet Street, London, UK; 5 Institute of Psychology, Humboldt-Universität Zu Berlin, Berlin, Germany; 6 Department of Child and Adolescent Psychiatry, Central Institute of Mental Health, Medical Faculty Mannheim, University of Heidelberg, Mannheim, Germany; 7 German Center for Mental Health (DZPG), Partner Site Mannheim-Heidelberg-Ulm, Berlin, Germany; 8 Department of Psychiatry, Brain Center Rudolf Magnus, University Medical Center Utrecht, Universiteitsweg 100, 3584 CG Utrecht, The Netherlands; 9 Department of Psychiatry, Autism Research Centre, University of Cambridge, Douglas House, 18B Trumpington Road, Cambridge, CB2 8AH UK; 10 Department of Psychology, The School of Philosophy, Psychology, and Language Sciences, University of Edinburgh, Dugald Stewart Building, 3 Charles Street, Edinburgh, EH8 9AD UK; 11 Donders Institute for Brain, Cognition and Behaviour, Radboud University Nijmegen Medical Centre, Kapittelweg 29, 6525 EN Nijmegen, The Netherlands; 12 Center for Neurodevelopmental Disorders at Karolinska Institutet (KIND), Stockholm, Sweden; 13 Human Genetics and Cognitive Functions Unit, Institut Pasteur, 25 Rue du Docteur Roux, Cedex 15, Paris, France; 14 Department of Child and Adolescent Psychiatry and Psychotherapy, Psychiatric Hospital, University of Zürich, Neumünsterallee 9, 8032 Zurich, Switzerland; 15 University Campus Bio-Medico, Via Álvaro del Portillo, 21, Rome, Italy; 16 Centre for Autism, School of Psychology and Clinical Language Sciences, University of Reading, Whiteknights, Reading, RG6 6AL UK; 17 Neurospin Centre CEA, 91191 SaclayGif Sur Yvette, France; 18 Roche Pharma Research and Early Development, Neuroscience, Ophthalmology and Rare Diseases, Roche Innovation Center Basel, Grenzacherstrasse 124, B.001 N.667, CH-4070 Basel, Switzerland; 19 Regulatory Affairs, Product Development, F. Hoffmann-La Roche Pharmaceuticals, Grenzacherstrasse 124, CH-4070 Basel, Switzerland; 20 Icahn School of Medicine at Mount Sinai, New York, NY USA; 21 Child and Youth Mental Health Collaborative, Centre for Addiction and Mental Health and The Hospital for Sick Children, Department of Psychiatry, University of Toronto, 80, Workman Way, Toronto, ON M6J 1H4 Canada; 22 Department of Psychology, Center for Applied Neuroscience, University of Cyprus, PO Box 20537, 1678 Nicosia, Cyprus; 23 Department of Psychiatry and Psychotherapy, Central Institute of Mental Health, Medical Faculty Mannheim, University of Heidelberg, 68159 Mannheim, Germany; 24 Child and Adolescent Neuropsychiatry Unit, Gaetano Martino University Hospital, University of Messina, Via Consolare Valeria 1, 98125 Messina, Italy; 25 Janssen Research & Development, 1125 Trenton Harbourton Road, Titusville, NJ 08560 USA

**Keywords:** Pupillometry, Autism spectrum condition, Mismatch negativity, Arousal, Predictive coding, Auditory oddball paradigm

## Abstract

**Background:**

Sensory processing requires selectivity to salient sensory input. Many autistic individuals report different sensory processing, which has been associated with altered sensory selectivity. The locus-coeruleus norepinephrine (LC-NE) system modulates the neuronal gain of sensory input, which represents a neurophysiological mechanism of sensory selectivity. In autistic individuals, we hypothesized that LC-NE tonic upregulation reduces sensory selectivity and underlies different sensory processing.

**Methods:**

Autistic (*n* = 139) and non-autistic (*n* = 98) individuals were assessed during a passive auditory oddball task with pupillometry and electroencephalography. For every trial, a baseline pupil size (BPS) assessed LC-NE tonic activity that coincides with current arousal, while a stimulus-evoked pupillary response (SEPR) assessed LC-NE phasic activity that estimated sensory selectivity. Electroencephalography assessed amplitudes of mismatch negativity (MMN-amp) that estimated pre-attentive change detection as a brain-activity readout of sensory selectivity. Measures were modeled between groups within the task by combining Frequentist and Bayesian approaches.

**Results:**

Across groups, higher BPS was associated with more negative MMN-amp to standards and oddballs. A more negative MMN-amp to standards was associated with a higher SEPR to standards. Controlling for these associations, autistic versus non-autistic individuals showed a higher SEPR in response to standards. In addition, a positive association of BPS and SEPR to standards was specific to autistic individuals. With task progression, autistic versus non-autistic individuals showed a higher initial increase and subsequently steeper decrease of BPS. This was supported by Bayesian posterior distribution estimates.

**Limitations:**

A short trial duration required concatenating trials to epochs and applying a linear-time invariant filter to capture the slow pupil changes. Without an LC-NE manipulation, we cannot rule out that pupil changes are evoked by other cortical pathways than the LC-NE.

**Conclusions:**

Across groups, LC-NE tonic upregulation is emphasized as a general mechanism that un-specifically increases pre-attentive change detection to all sensory stimuli, which then increases sensory selectivity to frequent stimuli. In autistic individuals, different sensory processing is characterized by increased sensory selectivity to frequent stimuli. This is likely caused by an LC-NE tonic upregulation. It associates autistic sensory processing with increased arousal upregulation that increases sensory selectivity to inconspicuous auditory information.

**Supplementary Information:**

The online version contains supplementary material available at 10.1186/s13229-025-00678-w.

## Background

Sensory processing describes the translation of sensory input to functional brain activity. Limited concurrent processing capacity requires the brain to select salient sensory input [1]. In bottom-up processing, the salience of sensory input is estimated by the conspicuity compared to competing information [2]. In top-down processing, the selectivity to salience is modulated by brain states like arousal [3] and expectations on the sensory input like predictions [4]. This adaptivity of selectivity is neurobiologically represented by changes in neuronal gain, which describes neurons’ input sensitivity [5]. The investigation of neuronal gain mechanisms might elucidate phenomena of altered sensory processing.

Autism spectrum disorder is a neurodevelopmental condition that is characterized by difficulties in social communication and restricted/repetitive behaviors. These characteristics have been considered as developmental adaptations to a different sensory processing [6]. The predictive coding account suggests a disrupted balance with increased bottom-up and decreased top-down (“priors”) sensory processing in autism [7]. The attenuated-priors model specified this to attenuated predictions reducing a sensory selectivity to salience [8]. This might explain autistic sensory processing with sensory hyperreactivity that contributes to sensory overloads and triggers compensatory behavior like insistence on sameness [9]. Here, we explore a neuronal gain mechanism and its contribution to altered sensory processing.

The locus-coeruleus norepinephrine (LC-NE) system is essential to sensory processing [10]. Under activation, the LC-NE proliferates norepinephrine (NE) via widespread projections [11]. NE release increases the signal-to-noise ratio in sensory processing cortices by inhibiting spontaneous neuronal activity and encouraging input-driven neuronal activity [12]. Thus, LC-NE activity is a mechanism of neuronal gain modulation [5, 13]. LC-NE phasic activity is a transient burst in frequency and amplitude, which emphasizes sensory selectivity to salient stimuli [14, 15] and is increased by top-down processes considering arousal and reward [16, 17]. LC-NE phasic activity distributes locally within functional LC-NE modules [11] that likely act cumulative in triggering a global sensory selectivity associated with attention [18, 19]. In contrast, LC-NE tonic activity has a variable, low frequency (1-5 Hz), which increases reactivity to all stimuli at the expense of sensory selectivity to salience [20] and corresponds to arousal levels [21, 22]. Arousal describes a global brain state of reactivity to all sensory information [23]. Thus, an upregulation of LC-NE tonic activity as elevated arousal might be associated with an attenuated sensory selectivity to salience in autism.

Video-based pupillometry has been shown to measure LC-NE activity [24]. In monkeys, LC-NE stimulation causes pupil size changes [25]. Baseline pupil sizes can be compared to estimate relative changes in LC-NE tonic activity [26]. In contrast, the stimulus-evoked pupillary response (SEPR) estimates LC-NE phasic activity [27]. In a probabilistic learning task, non-autistic but not autistic adults showed a larger SEPR for unexpected versus expected stimuli [28]. In an auditory oddball task, non-autistic but not autistic children showed an SEPR decline to frequent stimuli [29]. These findings indicate an attenuated sensory selectivity to salience in autism that might be driven by an increased reactivity to inconspicuous (i.e.: non-salient) sensory information. LC-NE tonic upregulation is a promising underlying mechanism as it increases reactivity at the expense of sensory selectivity [21]. We investigate such changes of tonic LC-NE activity in an auditory oddball task and its effects on sensory selectivity.

Mismatch negativity (MMN) assesses pre-attentive change detection in the auditory oddball task [30, 31]. It represents an event-related potential in electroencephalography to assess early stages of sensory selectivity [32]. MMN is usually calculated as an amplitude response difference to infrequent (oddball) versus frequent stimuli (standards) [33]. Autistic compared to non-autistic individuals showed reduced MMN in non-speech auditory oddball tasks [34]. This could be caused by altered amplitudes to both oddballs or standards. In autistic children, an attenuated neurophysiological amplitude decline when listening to sequences of standards compared to non-autistic children has been interpreted as attenuated sensory habituation [35, 36]. In autism, a higher response to standards might mitigate the response difference to oddballs and explain a reduced MMN as attenuated pre-attentive change detection. We expect that the MMN—as an early marker of sensory selectivity to salience—functionally relates to neuronal gain [37] and is thus modulated by LC-NE tonic activity [5].

We investigate LC-NE tonic activity as a mechanism of altered sensory processing in autism. We assess autistic and non-autistic individuals in a passive auditory oddball task. We utilize baseline pupil size (BPS) to estimate LC-NE tonic activity that is modelled with task progression to assess changes in sensory processing. We further investigate effects of LC-NE tonic activity on different stages of sensory processing including MMN-associated amplitude as an index of early change detection and SEPR as index of later sensory selectivity. In autistic versus non-autistic individuals, we expect BPS increases within the task, which would indicate LC-NE tonic upregulation. BPS increases will be related to the adaptation of change detection and sensory selectivity to characterize a mechanism of different sensory processing in autistic individuals.

## Methods

### Sample

The sample (autistic: *N* = 140; non-autistic: *N* = 98) was recruited at two sites (Mannheim, London, see Table S1) and is a subsample of the EU-AIMS LEAP study [38]. This includes all participants that were assessed with concurrent eye-tracking and electroencephalography (EEG). Inclusion and exclusion criteria are elaborated in the clinical characterization of EU-AIMS LEAP, while the procedure of group assignment included gold standard diagnostics [38]. For our analysis, groups were matched based on initial differences in perceptual IQ and task attendance (see Table 1, for distributions Fig. S1). We analyzed the eye-tracking data of two timepoints (baseline, follow-up after 12–24 months), while EEG was available at baseline.

**Table 1:** Sample description

	Autistic individuals	Non-autistic individuals	Group diff. (p)
n	140	98	–
Gender (M/F)	105/35	61/37	0.049
Timepoints (1/2/1 + 2)	41/48/51	27/36/35	0.92
Age (in years)	16.15/5.51 [6.24–29.23]	17.43/5.96 [6.24–30.98]	0.093
IQ	98.32/21.18 [46–148]	104.51/21.12 [50–142]	0.028
Perceptual IQ	99.58/21.05 [46–138]	104.19/20.96 [49–147]	0.097
Verbal IQ	96.58/20.7 [45–160]	103.3/20.92 [51–160]	0.016
Missing data per trial (%)	15.89/17.17 [0.77–99.98]	13.19/14.02 [0.41–73.94]	0.184
Sampling rate (300 Hz / 120 Hz)	127/72	89/44	0.644
Gaze center deviation (%)	18.33/2.43 [13.39–26.9]	17.89/2.3 [13.42–25.79]	0.157
Mean pupil size (mm)	3.52/0.51 [2.32–4.82]	3.36/0.55 [2.28–5.9]	0.023
Medication (yes/no)	65/75	20/78	< 0.001
SRS (total)	100.47/29.75 [20–168]	30.58/30.67 [1–113]	< 0.001
RBS (total)	20.67/16.08 [0–90]	2.93/5.97 [0–34]	< 0.001
SDQ (total)	17.69/6.4 [3–34]	9.3/8.24 [0–30]	< 0.001
ADHD inattention	5.18/3.25 [0–9]	1.78/2.72 [0–9]	< 0.001
ADHD hyperactivity	3.24/3.02 [0–9]	1/2.03 [0–9]	< 0.001
BAI (anxiety)	15.26/11.36 [0–63]	9.58/8.27 [0–38]	< 0.001
BDI (depression)	13.64/11.44 [0–61]	9.05/9.31 [0–39]	< 0.001

Total numbers are provided for individuals (n), gender, medication, and timepoints. Other variables are described by the statistics: mean / standard deviation [min – max]. Supplementary Note 1 provides further information on the type of medication. perceptual IQ = a non-verbal IQ estimate based on the block design and matrix reasoning subtests of the age-appropriate Wechsler Intelligence Scale (WAIS-IV, WISC-IV); verbal IQ = a verbal IQ estimate based on the similarities and vocabulary subtests of the age-appropriate Wechsler Intelligence Scale (WAIS-IV, WISC-IV); SRS = Social Responsiveness Scale; RBS = Repetitive Behavior Scale; SDQ = Strengths and Difficulties Questionnaire; ADHD = ADHD rating scale DSM-5; BAI = Beck’s Anxiety Inventory; BDI = Beck’s Depression Inventory. Further information on all measures is provided in the clinical characterization of the EU-AIMS LEAP data set [38].

### Procedure

**Fig. 1:**
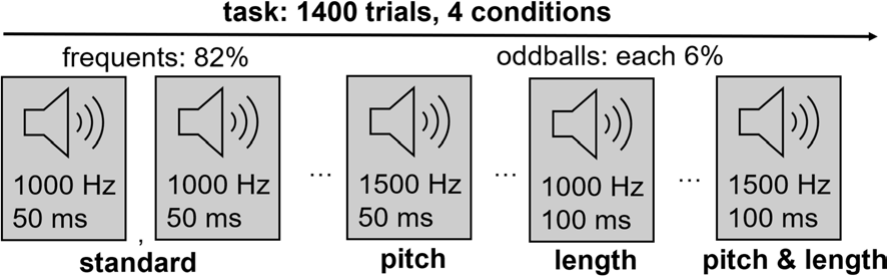
Task description of the passive auditory oddball task. The auditory oddball tasks include the four conditions of standards, pitch oddballs, length oddballs, and pitch & length oddballs

Participants were presented with a passive auditory oddball task, while eye-tracking and EEG was recorded. The task was presented in one block along with a silent cartoon. Participants were instructed to watch the cartoon and encouraged to attend when attention waned. The task took 14 min to complete. It consisted of 1400 trials in a pseudorandomized sequence of frequent pure tones (inconspicuous standards) and three conditions of infrequent—and thus salient—oddballs (1 tone = 1 trial, Fig. 1). The three oddball conditions were originally implemented to investigate differences in MMN responses towards pitch versus length oddballs but are secondary to the current research objective. Oddball trials were never presented consecutively. Each trial had a random inter-stimulus-interval between 500 and 600 ms. Pure tones were presented on speakers next to the presentation screen leveled at 70db. The presentation screens were 17- or 23-inch displays with a fixed display area of 345 × 259 mm. The procedure was carried out with controlled artificial lights adapted for optimal eye detection (lux: m/SD = 148/156) and without a chin rest. Artificial lighting did not differ between groups or sites (*t* < 1).

Eye-tracking was applied to record pupillometry and monitor screen attention. The eye-trackers were a Tobii X-120 (120 Hz sampling rate, London) and Tobii TX-300 (300 Hz sampling rate, Mannheim) with a target eye-to-screen distance of 65 cm. Tobii eye-trackers estimate pupil diameter based on a 3D-eye-model scaling factor, which considers parallax effects on pupil size estimation. Electroencephalography (EEG) was recorded using 64 Ag/AgCl electrodes (Electrical Geodesics Inc.) and BrainAmp amplifiers. A gel solution was applied to improve conduction. Impedance was kept below 20kΩ and no online filter was applied.

### Data preprocessing

#### Pupillometry

Pupil data were preprocessed with R statistics (4.3) according to peer-reviewed guidelines [39] for each trial with an exclusion of impossible diameters (< 2 mm, > 8 mm), linear filtering (< 3 times median absolute deviation), blink correction (25 ms before and after, 75–250 ms blinks), and outlier deletion with missing interpolation (150 ms window). The estimated pupil size was based on the mean of both eyes (r = 0.98).

Pupillary responses are slow with a response curve that peaks around 1 s and returns to baseline after 2–4 s [29, 40, 41]. Thus, we combined data of each trial (500-600 ms) with the data of the next 7 trials to generate epochs (epoch duration = 4 – 4.8 s). We investigated the pupillary response to the first trial in each epoch. This required to filter out the noise generated by the pupillary responses related to the 7 subsequent trials within an epoch. For this, we adapted a linear time-invariant (LTI) filtering technique from rapid-event related designs established in functional magnet-resonance imaging [42]. We estimated a canonical pupillary response function (CPF) as a gamma-distribution (shape = 6.65 [6.47, 6.82], rate = 7.68 [7.47, 7.89]) with non-linear least square optimization (Fig. S2, fit: R^2^ = 0.69). To avoid overfitting, this CPF was estimated in an independent dataset (*n* = 118, each with 400 trials) of a passive auditory oddball task with longer trial durations (1800–2000 ms) in a sample of autistic and non-autistic adolescents (age: m/SD = 15.4/1.9) with average cognitive ability (perceptual IQ: m/SD = 104/12). The longer trial duration in the independent dataset ensured that the CPF estimation is less affected by overlapping signals compared to the current dataset. Each epoch was LTI-filtered by subtracting the CPF related to the 7 subsequent trials (Fig. S3). The CPF is scaled in amplitude according to the trial type (standards = 0.001, oddballs = 0.01) to approximate expected pupillary response magnitudes.

The baseline pupil size (BPS) was calculated as a mean pupil size during each filtered epoch that was additionally corrected for the CPF of the first trial. The BPS represents a residual pupil size that is corrected for the pupillary responses to stimuli. All pupil size estimates were normalized across epochs by subtracting the respective BPS [43]. For each epoch, the stimulus-evoked pupillary response (SEPR) was estimated as a mean of the normalized pupil size between 0.75 and 1.75 s, which corresponds to a pupil size change in response to the first trial within an epoch. An alternative SEPR as change velocity did not alter results (task or group effects) compared to SEPR as mean and, thus, was not further considered.

The two timepoints allowed to assess test–retest reliability for BPS with ICC = 0.82 [0.73, 0.88] and SEPR with ICC = 0.27 [0.06, 0.46] (for scatterplots see Fig. S4). Pupillometry preprocessing retained BPS in 91.2% of all possible trials (autistic = 89.7%, non-autistic = 95.2%, *χ*^2^(1) = 629.88, *p* < 0.001) and SEPR in 89.9% of all trials (autistic = 87.6%, non-autistic = 93.4%, *χ*^2^(1) = 670.14, *p* < 0.001).

Pupil size is largely dependent on environmental luminance. Thus, we estimated the relative luminance of the cartoon that was presented during the oddball task. For each video frame, RGB values were linearized by inverting a gamma correction of 2.2 and converted to a relative luminance with the sRGB coefficient (= 0.2126 * R + 0.7152 * G + 0.0722 * B). This relative luminance (Fig. S5) was applied as a covariate in the BPS and SEPR models. Pupil size estimates are also dependent on data quality. This was considered with variables of missing data and gaze center deviation in the models (see below statistical analysis). We investigated these variables with task progression, which indicated higher missing data in autistic individuals, but no systematic effects between groups with task progression (Figs. S6, S7).

#### Electroencephalography

EEG data were initially preprocessed by the EU-AIMS LEAP consortium by excluding incomplete and noisy datasets [44]. EEG data were further preprocessed with Matlab’s (2016a) EEGLAB toolbox [45]. This included re-referencing to the FCz-electrode, manual identification, and interpolation of noisy and flat channels with continuous data rejection. Data were down-sampled to 1000 Hz and filtered with a 1-30 Hz Hamming window finite impulse response (FIR) filter for an Adaptive Mixture Independent Component Analysis (ICA) with shared components [46]. The number of extracted components was reduced by the number of channels which had been interpolated. Eye blinks and saccadic ICA components were removed manually based on two raters’ agreement. Acquired ICA weights were transferred to the event-related potential (ERP) analysis, which was filtered with a 0.1–30 Hz Hamming window FIR filter [47]. Data were segmented from − 100 ms to 500 ms after trial onset. An automated artifact rejection (- + 100 μV) was performed.

A MMN-associated amplitude in each trial was defined as the mean negative peak of the Fz channel of 20 ms sliding intervals within 50–350 ms [34]. We applied the MMN-associated amplitude to analyze group differences with task progression within conditions compared to a difference measure between conditions. Per participant, MMN preprocessing retained m = 62.71 (SD = 10.30) trials of an oddball condition, which translates to 74.6% of possible trials per condition. To balance the number of trials between conditions per participant, the lowest number of retained trials across conditions was applied to select trials in all conditions. MMN-associated amplitude was retrieved in 75% of autistic and 74% of non-autistic individuals (*χ*^2^(1) < 1).

#### Statistical analysis

Analysis scripts are available online. Dependent variables were pupillometry measures (BPS, SEPR), and mismatch-negativity-associated amplitude (MMN-amp, data distribution: Figs. S8 and 9). Variables were z-standardized between participants. Group differences *across the task* were investigated for aggregated measures with linear fixed-effect models. Association of measures across the task were investigated with two-tailed Pearson correlation with multiple comparison correction (Bonferroni correction: *p*-value × 12 comparisons). Significant correlations were further characterized between groups with linear fixed-effect models.

Changes *within the task* between groups were investigated by applying linear mixed models to BPS, SEPR, and MMN-amp. The linear mixed models applied restricted maximum likelihood estimation (REML), whereas model comparisons were refit with ML. Participant was a random intercept in all models. First, an association analysis applied the dependent variables (BPS, SEPR, MMN-amp) as single predictors of each other. Next, task effects on BPS and SEPR and MMN-amp were independently explored with fixed effects of stimulus (standard vs. pitch oddball vs. length oddball vs. pitch & length oddball) or task progression (trial: 1–1400). Last, group comparisons were fitted with group (autistic vs. non-autistic) as fixed effect and interactions between all fixed effects. This interaction (group x stimulus x task progression) estimated changes of BPS, SEPR, and MMN-amp within the task between groups. BPS and SEPR models included relative mean luminance of the current stimulus material in that trial as a covariate. In supporting models, we controlled group differences for the covariates of age, perceptual IQ, gender, sampling rate, data quality, and gaze center deviation. Site effects are effectively controlled via sampling rate (see procedure).

Model fits were estimated with coefficient of determination (R^2^) for linear models and the marginalized (*mR*^2^) and conditional (*cR*^*2*^) coefficient of determination for linear mixed models [48]. Fixed effect significance was estimated by ANOVA using Satterthwaite’s method [49]. Given the approximative nature of estimating degrees of freedom for effect significance in linear mixed models, effect evidence was estimated by the BIC-derived Bayes Factor (*BF*) to support effect significance in linear mixed models [50]. In linear mixed models, we only interpreted significant effects with effect evidence of BF > 1. Fixed effects were reported as standardized coefficients (β) and 95% bootstrapped confidence intervals. Interactions were investigated post-hoc with contrasts family-wise corrected for multiple comparisons (Tukey HSD) with marginalized means (Δβ) and 95% bootstrapped confidence intervals.

We applied Bayesian modelling to cross-validate group differences observed in linear mixed models [51] by a TensorFlow implementation [52]. In a Bayesian hierarchical random intercept model with normal distribution priors, a mean response was defined as a function of additive fixed effects, the fixed-effect interaction with group, and a random intercept for participant. We modeled a likelihood distribution as a normal distribution of the mean response with a standard deviation as a Cauchy distribution (location = 0, scale = 3). Posterior sampling was estimated by 4 Monte Carlo Markov chains with 4000 warmups and 8000 iterations. Convergence and stationarity are presented in Fig. S10. Posterior distribution estimates (b) are presented with 89% credible intervals [53].

## Results

Group differences across the task: Baseline pupil size (BPS), stimulus-evoked pupillary response (SEPR), and mismatch-negativity-associated amplitude (MMN-amp).

**Fig. 2:**
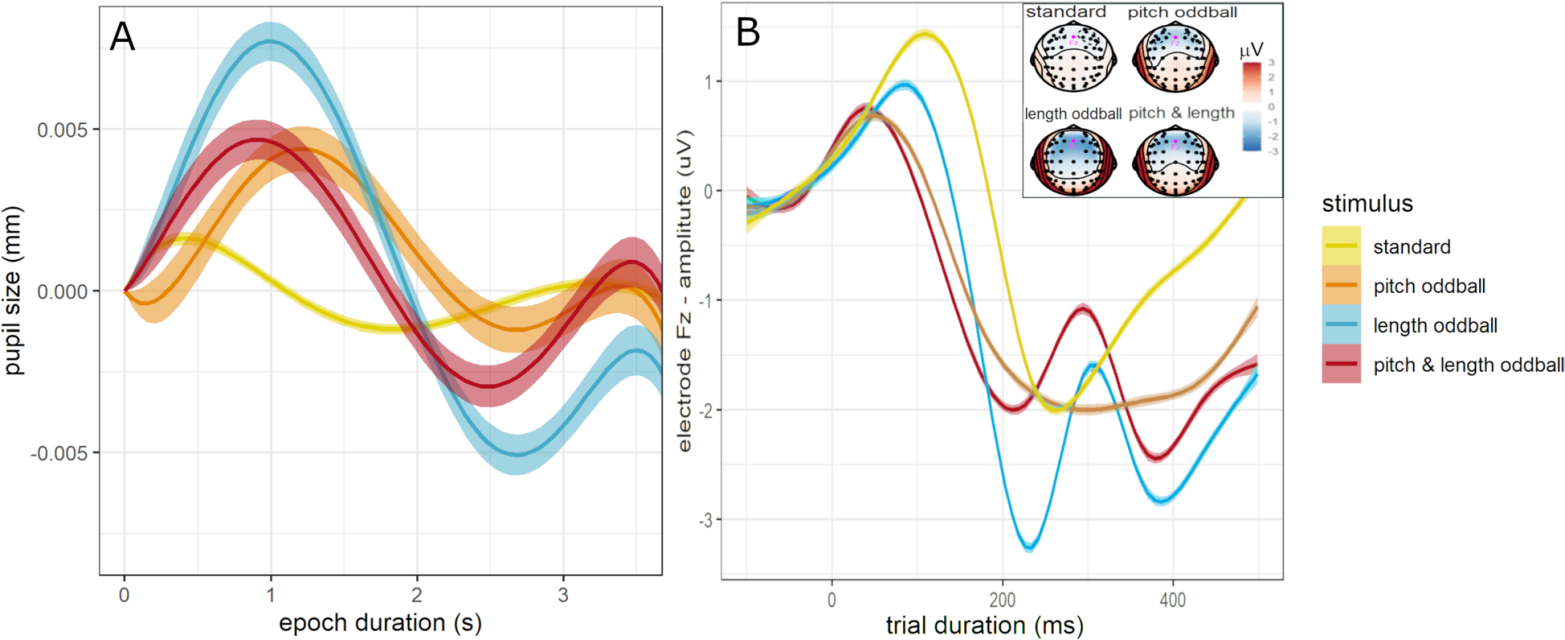
Dependent variables are calculated by pupil size changes (BPS, SEPR) and Fz-electrode amplitude changes (MMN-amp) within trials. **A**. Pupil size change within epoch by stimulus (shaded area = 95% CI), which was applied to estimate baseline pupil size (BPS, mean of epoch corrected for all stimulus effect, see data preprocessing) and stimulus-evoked pupillary response (SEPR, mean of epoch between 750 and 1750 ms retaining the stimulus effect of the first trial). **B**. Amplitude changes of the Fz electrode within trials were applied to estimate MMN-associated amplitude (negative peak between 50 and 350 ms, shaded area = 95% CI) similarly across and within the task. Inlay: Scalp topography of electrode amplitudes between stimuli within 150-250 ms after stimulus onset. The Fz electrode marked in magenta was utilized to estimate MMN-associated amplitude

Changes of pupil size and electrode amplitude within trials were utilized to calculate BPS, SEPR, and MMN-amp (Fig. 2). Descriptive statistics and full covariate models are provided in Table S2–S5. Medication differed between groups and did not alter group differences reported below (see also Supplementary Note 1).

Across the task, BPS was higher in autistic versus non-autistic individuals (*F*(1,329) = 6.64, *p* = 0.001, *R*^2^ = 0.02, *d* = 0.29 [0.07, 0.51]). Inclusion of the covariates age and gaze center deviation (data quality) moderated the group difference in the aggregated BPS (*F*(1,327) = 3.05, *p* = 0.082, Table S5). Covariate effects were characterized by a decrease of BPS with age (β = − 0.38 [-0.47, − 0.29]) and increase of BPS with higher gaze center deviation (β = 0.18 [0.08, 0.26]) across groups. Descriptive associations of BPS, SEPR, and MMN-amp with age, IQ, and biological sex are shown in Figs. S11–S13.

### Associations of BPS, SEPR, and MMN-amp

Across groups, significant correlations associated a higher BPS with a higher SEPR to standards (*r*(329) = 0.16 [0.06, 0.27]) and length oddballs (*r*(329) = 0.17 [0.06, 0.27]; Table S6). A higher BPS was further associated with a more negative MMN-amp to standards (*r*(254) = − 0.18 [− 0.29, -0.06]) and oddball stimuli (pitch: *r*(253) = − 0.19 [− 0.31, − 0.07]; length: *r*(253) = − 0.21 [− 0.33, − 0.09]; pitch & length: *r*(253) = − 0.21 [− 0.33, − 0.09]). A more negative MMN-amp to standards was associated with a higher SEPR to standards (*r*(254) = − 0.20 [− 0.31, − 0.08]).

Significant correlations were further investigated between groups in linear regression models with group as fixed effect. For SEPR to standards as dependent variable and BPS as predictor (*R*^2^ = 0.04, Table S7), a significant interaction with group (*F*(1,327) = 4.49, *p* = 0.035) showed that the positive association was specific to autistic (β = 0.25 [0.11, 0.39]) compared to non-autistic individuals (β = 0.02 [− 0.14, 0.18]). For SEPR to standards as dependent variable and MMN-amp to standards as predictor (*R*^2^ = 0.06, Table S8), a significant main effect of group emerged (*F*(1,252) = 5.68, *p* = 0.017) that was characterized by a higher SEPR to standards in autistic compared to non-autistic individuals (Δβ = 0.27 [0.05, 0.48]). Other significant associations mimicked the correlations above and were not influenced by group.

### Stimulus effects across groups

**Fig. 3:**
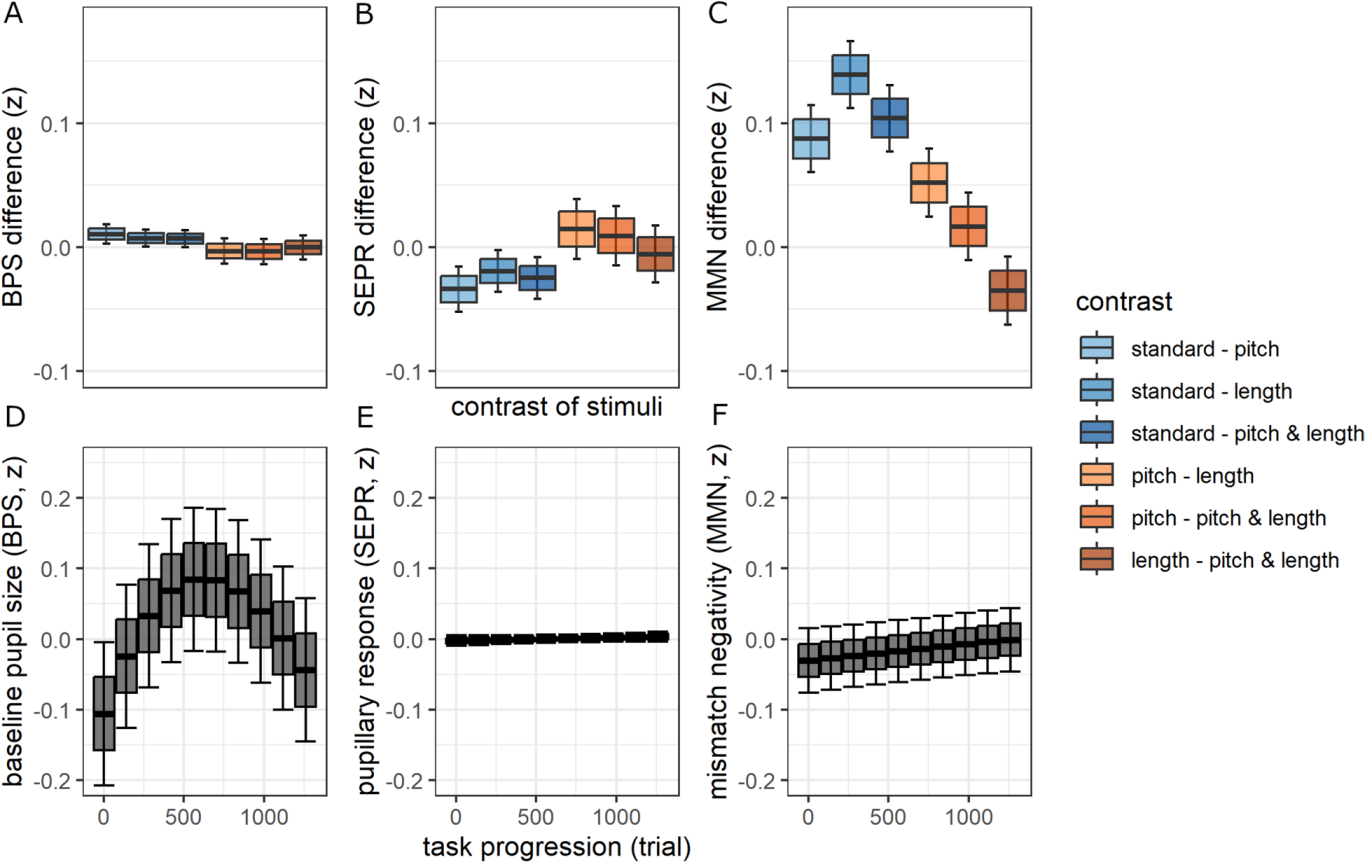
Effect of stimulus and task progression across groups. Effect of stimulus (**A**–**C**) and task progression (**D**–**F**) on baseline pupil size (BPS, left), stimulus-evoked pupillary response (SEPR, middle), and mismatch-negativity-associated amplitude (MMN-amp, right) across groups. All variables were z-standardized (z)

The following analyses apply linear mixed models on a per-trial level compared to the linear fixed models on a per-participant level above. Figure S14 provides data point distributions on a per-trial level. Bayes Factor (BF) is applied as effect evidence to support effect significance. BPS did not differ by stimulus based on effect evidence (*p* < 0.001, *BF* < *1*). SEPR differed by stimulus with moderate evidence (*p* < 0.001, *BF* = 3.1). Post-hoc analysis showed that SEPR was lower for standard trials compared to pitch (Δβ = − 0.03 [− 0.05, − 0.02]), length (Δβ = − 0.02 [− 0.04, 0.00]), and pitch & length (Δβ = − 0.02 [− 0.04, − 0.01]) oddballs (Fig. 3B). MMN-amp differed by stimulus with strong evidence (p < 0.001, *BF* > 100). Post-hoc analysis showed a less negative MMN-amp for standard trials compared to pitch (Δβ = 0.08 [0.05, 0.11]), length (Δβ = 0.14 [0.11, 0.17]), and pitch & length (Δβ = 0.10 [0.08, 0.13]) oddballs. MMN-amp was also less negative in pitch compared to length oddballs (Δβ = 0.05 [0.02, 0.08]) (Fig. 3C).

### Changes within the task across groups

Changes of the dependent variables within the task were modeled by comparing different polynomial fits for task progression (Table S9). Here, we report the best fit models. BPS changed cubically with task progression (*p* < 0.001, *BF* > 100, *mR*^2^ = 0.01, *cR*^*2*^ = 0.77), which translates to a BPS increase in the first half (trial 700 vs 1, Δβ = 0.19 [0.18, 0.20]) and a (marginally attenuated) BPS decrease in the second half (trial 1400 vs 700, Δβ = − 0.18 [− 0.19, -0.17], Fig. 3D). SEPR (*p* = 0.063) and MMN (*p* = 0.010) did not change with task progression based on effect evidence (*BFs* < 1, Fig. 3E, F).

### Group differences in changes of BPS, SEPR, and MMN-amp

**Fig. 4:**
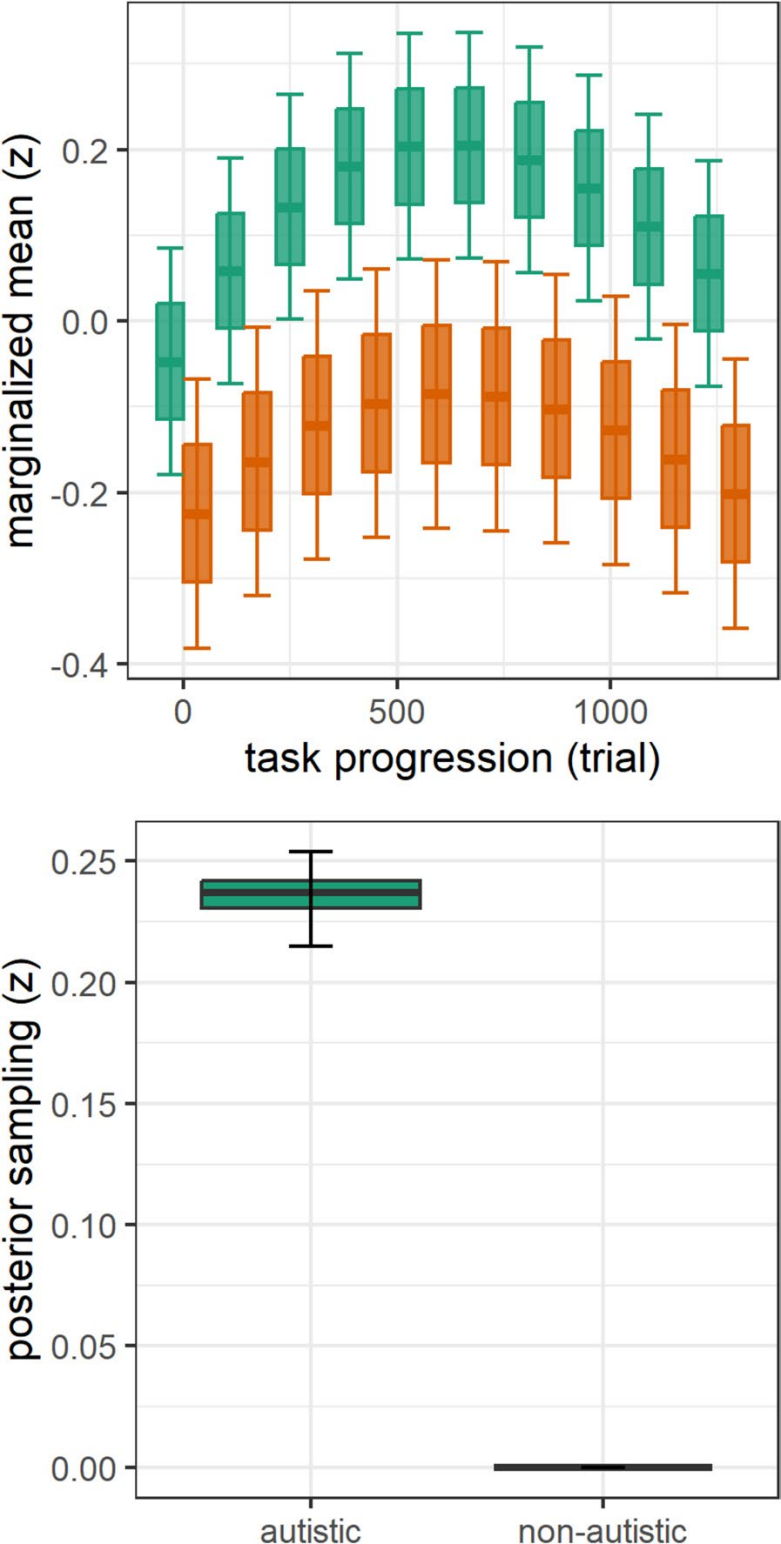
Group differences of changes in baseline pupil size (BPS). Group differences in BPS with progression of the oddball task by stimulus between autistic (green) and non-autistic (orange) individuals. Top panels show estimated means of linear mixed models; boxplots indicate a + / − 1 standard-error margin and 95% confidence interval of the estimated means for binned trials (each 10%). Bottom panels show posterior distribution estimates of Bayesian sampling; boxplots plots indicate the interquartile range and 89% credible intervals. All variables were z-standardized (z)

For the cubic-fit task progression model (*mR*^2^ = 0.02, *cR*^*2*^ = 0.83, Fig. 4), BPS was characterized by an interaction of group x task progression (*F*(3,426,950) = 42.13, *p* < 0.001, *BF* > 100), which was not moderated by including covariates (*F*(3,425,393) = 47.16, *p* < 0.001, BF > 100, Table S10). Post-hoc analysis showed a higher BPS increase in the first half of the task (trial 700 vs 1) in autistic (Δβ = 0.25 [0.24, 0.27]) compared to non-autistic individuals (Δβ = 0.14 [0.12, 0.16], as well as a steeper BPS decrease in the second half of the task (trial 1400 vs 700) in autistic (Δβ = − 0.21 [− 0.23, -0.19]) compared to non-autistic individuals (Δβ = − 0.16 [− 0.18, − 0.14]). This was supported by Bayesian posterior estimates that showed a BPS increase in autistic individuals (*b* = 0.24 [0.21, 0.25]) compared to non-autistic individuals (*b* = 0.00 [0.00, 0.00]). For the linear-fit task progression model (*mR*^2^ = 0.00, *cR*^*2*^ = 0.00, Fig. 4B), SEPR did not differ between groups (*p* = 0.537, *BF* < 1). For the linear-fit task progression model (*mR*^2^ = 0.00, *cR*^*2*^ = 0.13), MMN-amp was characterized by an interaction of group x stimulus x task progression (*F(*3,64,124) = 3.97, *p* = 0.007). However, the effect evidence was low (*BF* < 1).

## Discussion

The study aimed to characterize sensory processing in neurodiversity. We combined pupillometry with electroencephalography in a passive auditory oddball task to specify a mechanism of sensory processing that differs between autistic and non-autistic individuals. In autism, our findings support LC-NE tonic upregulation within the task as a mechanism of neuronal gain modulation that increases selectivity to inconspicuous auditory information.

Sensory processing entails an increased selectivity to conspicuous (i.e. salient) stimuli [1]. MMN has been established as pre-attentive change detection marking conspicuous stimuli [30]. Our findings showed a more negative MMN-amp as well as an increased SEPR for oddballs compared to standard stimuli across groups. It supports MMN-amp as an early marker and SEPR as a later marker of sensory selectivity within sensory processing that are both reactive to the salience of the sensory input. As expected, BPS was not modulated by stimulus salience (see Fig. 3). This supports BPS as slowly changing LC-NE tonic activity that is not immediately reactive to the current sensory input.

Across groups, higher BPS was associated with more negative MMN-amp across standard and oddball stimuli. It indicates that increased LC-NE tonic activity unselectively increases pre-attentive change detection to all sensory stimuli. Further, a more negative MMN-amp to standards was associated with a higher SEPR to standards. LC-NE tonic activity has also been associated with general arousal levels [21, 23]. Thus, arousal upregulation likely decreases a neurophysiological signal-to-noise ratio in differentiating between salient oddball and non-salient standards by increasing neuronal gain to inconspicuous standards [23, 54].

The associations between indices of arousal (BPS), pre-attentive change detection (MMN-amp), and sensory-driven selectivity (SEPR) differed in autistic versus non-autistic individuals. In autistic but not non-autistic individuals, a higher BPS was associated with an increased SEPR to standards. In addition, such an increased SEPR to standards was observed when controlling for the effect of MMN-amp on SEPR for standards. Autistic individuals showed increased LC-NE tonic upregulation in a basic sensory processing task that contributed to a higher sensory-driven selectivity to standards. This might underlie an overestimation of volatility in sensory environments [28]. We conclude that sensory processing of autistic individuals in the auditory domain associates increased arousal with an increased reactivity to inconspicuous stimuli. This could result in sensory hyperreactivity that is often reported as sensory experience in autistic individuals [9].

Across the task, we observed a higher mean BPS in autistic compared to non-autistic individuals, which contributes to a heterogenous literature (*I*^2^ = 66.2%) on mean BPS differences in autism [55]. Covariate effects indicate that this mean BPS difference could be specific to younger age and less attentive participants (Table S5). Covariate associations with BPS further indicate that higher perceptual IQ might be associated with increased BPS differences across groups (Fig. S11). Overall, we conclude that sensory processing in autistic individuals is characterized by increased arousal during auditory sensory processing.

Importantly, this higher mean BPS was specified by group-specific changes within the task. Both groups show an inverted-U-shaped change of BPS with task progression, which indicates that LC-NE tonic activity initially increases but wanes with task progression (Fig. 3D). However, autistic versus non-autistic individuals showed a higher initial BPS increase and subsequently a higher BPS decrease towards the end of the task. This is supported by Bayesian posterior estimates that showed increasing BPS with task progression in autistic versus non-autistic individuals. We conclude that autistic versus non-autistic individuals experience an arousal upregulation during auditory sensory processing that contributes to an increased sensory selectivity to inconspicuous standards. This increased arousal in autistic individuals during sensory processing likely contributes to an attenuated neurophysiological habituation to standards [35, 36] and might be explained by LC-NE tonic upregulation.

Vice versa, previous findings in autistic individuals of an attenuated reactivity to conspicuous (salient) versus inconspicuous (non-salient) sensory information [34] might be driven by the increased change detection of and selectivity to inconspicuous sensory information. Previous computational models of LC-NE functioning combined with neuroimaging data separated processes of arousal-associated amplification of conspicuous sensory information versus suppression of inconspicuous information [54]. Our findings support a mechanism of LC-NE tonic upregulation that curtails the suppression of inconspicuous information in autistic individuals. This also entails phenotypic similarities with aging-associated changes in LC-NE functioning [54]. Future studies might explore how the increasing literature of LC-NE functioning in neurodegenerative conditions [56] translates to sensory processing in neurodiversity.

As a final perspective, we argue that the current findings also inform the predictive coding framework in autism [7]. In this model, autistic individuals are suggested to overestimate the reliability of sensory input (“increased precision weighting”) [6] that inflates prediction errors for non-salient information and might explain sensory hyperreactivity [8]. This has been supported by a computational model, in which autistic versus non-autistic showed elevated prediction errors that were measure as pupillary responses [28]. We argue that LC-NE tonic upregulation associated with pupil size changes during sensory processing provides a promising mechanism of increased precision weighting that inflates prediction errors [57]. This is further supported by a recent meta-analysis that emphasized MMN as an established measure of prediction errors, which was modulated in our analysis by LC-NE tonic activity [58]. We propose that sensory processing in autism is characterized by an increased upregulation of LC-NE tonic activity that elevates neuronal gain to standards and embodies increased precision weighting in predictive coding.

## Limitations

Our findings are limited by a short trial duration that was not designed to capture relatively slow pupil changes. This has been addressed by concatenating trials to epochs of sufficient length and correcting the stimulus-associated pupillary responses within the epoch by an independently derived LTI filter. The resulting BPS was thus corrected for stimulus-associated responses and hence not modulated by stimulus type, whereas the resulting SEPR included the stimulus-associated response to the first trial and was modulated by stimulus type. It provides evidence that an LTI-filtering of pupil data was able to control for stimulus-induced changes (Fig. S3). However, we were not able to replicate a lower SEPR to oddballs in autistic versus non-autistic adolescents that has been reported in a smaller sample for visual oddballs [41]. In addition, we outline that other cortical pathways independent of LC-NE activity could contribute to pupil size changes [59, 60] and cannot be differentiated based on the current analysis. Lastly, the group-based matching based on perceptual IQ led to an autistic sample with a higher mean perceptual IQ than the autistic population, and thus reduces the generalizability of observed findings.

## Conclusions

The combination of pupillometry (BPS, SEPR) and EEG (MMN-amp) has been applied to specify LC-NE tonic upregulation (BPS) as a promising underlying mechanism of altered sensory processing in autistic versus non-autistic individuals. Future studies may include an experimental manipulation of LC-NE activity [61] to investigate whether experimentally induced changes in LC-NE tonic activity provoke a modulation of pre-attentive change detection (MMN-amp) and sensory-driven selectivity (SEPR). Together, we conclude that LC-NE tonic upregulation during sensory processing increases sensory-driven selectivity to inconspicuous auditory information, which may contribute to symptoms of sensory hyperreactivity in neurodiversity.

## Supplementary Information

Supplementary Information 1.

## Data Availability

EU-AIMS LEAP data are shared through an application process codesigned with autistic people that preserves security and privacy—contact the corresponding author for further details and application forms. Scripts used to implement the experimental task are covered by a material transfer agreement, which can be obtained through the corresponding author. The data analysis scripts are accessible online ([https://github.com/nicobast/oddball_LEAP]).

## Abbreviations

BPS: Baseline pupil size
CPF: Canonical pupillary response function
FIR: Finite impulse response
ICA: Independent component analysis
LC-NE: Locus-coeruleus norepinephrine
LTI: Linear time-invariant
MMN: Mismatch negativity
MMN-amp: Mismatch-negativity associated amplitude
REML: Restricted maximum likelihood
SEPR: Stimulus-evoked pupillary response
