# Supplementary material for: Locus coeruleus tonic upregulation increases selectivity to inconspicuous auditory information in autistic compared to non-autistic individuals: a combined pupillometry and electroencephalography study
Source: Mol Autism. 2025 Aug 21;16:41. doi: 10.1186/s13229-025-00678-w (PMC12372287; doi:10.1186/s13229-025-00678-w)
Supplement: Supplementary file 1 — Supplementary Information 1. [file 13229_2025_678_MOESM1_ESM.pdf]

Supplementary information

Table of Content

Figure S1 – histograms of participant characteristics between groups 2
Figure S2 – canonical pupillary response function 3
Figure S3 – Sample trial of LTI-filtered pupillary response 4
Figure S4 – scatterplot of BPS and SEPR between timepoints 5
Figure S5 – relative luminance across the task 6
Figure S6 – data quality as missing data with task progression between groups 7
Figure S7 – data quality as gaze center deviation with task progression between groups 8
Figure S8 - histograms of per-participant estimates 9
Figure S9 - histograms of per-trial estimates 10
Figure S10 - Bayesian posterior estimate MCMC chain convergence 11
Figure S11 - BPS association with age, IQ, biological sex 12
Figure S12 - SEPR association with age, IQ, biological sex 13
Figure S13 - MMN-amp association with age, IQ, biological sex 14
Figure S14 – data point distributions between stimulus conditions and with task progression .. 15
Table S1 – descriptive statistics between sites 16
Table S2 - descriptive statistics on participant level 17
Table S3 - descriptive statistics on trial level 18
Table S4 - group comparisons on per-participant estimates 19
Table S5 - covariate effects: BPS group difference 20
Table S6 - correlation table of BPS, SEPR, MMN-amp, NG 21
Table S7 – SEPR regression model with BPS as predictor between groups 22
Table S8 – SEPR regression model with MMN as predictor between groups 23
Table S9 - polynomial fit comparison for BPS, SEPR; MMN-amp 24
Table S10 - BPS linear mixed model - task progression 25
Supplementary Note 1 - effect of medication on group differences 26

Figure S1 – histograms of participant characteristics between groups

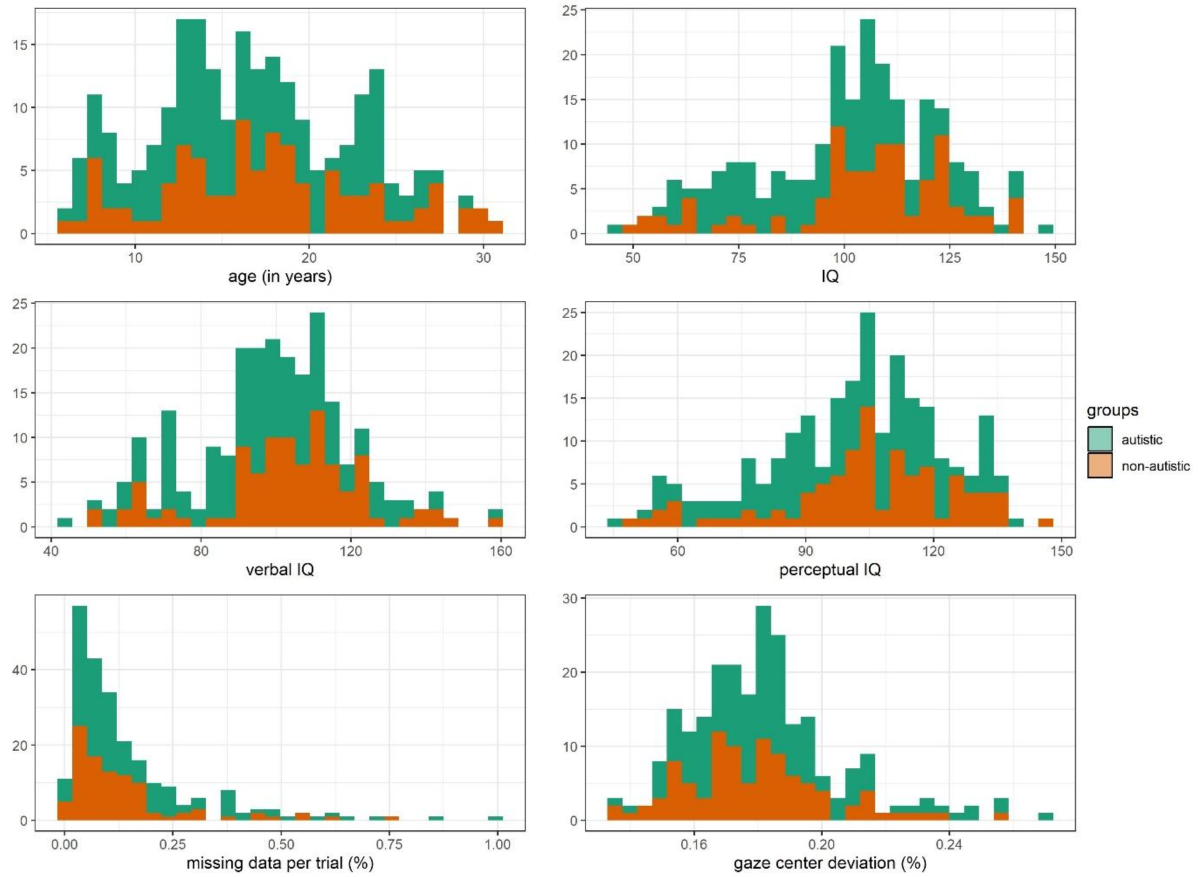

Figure S2 – canonical pupillary response function

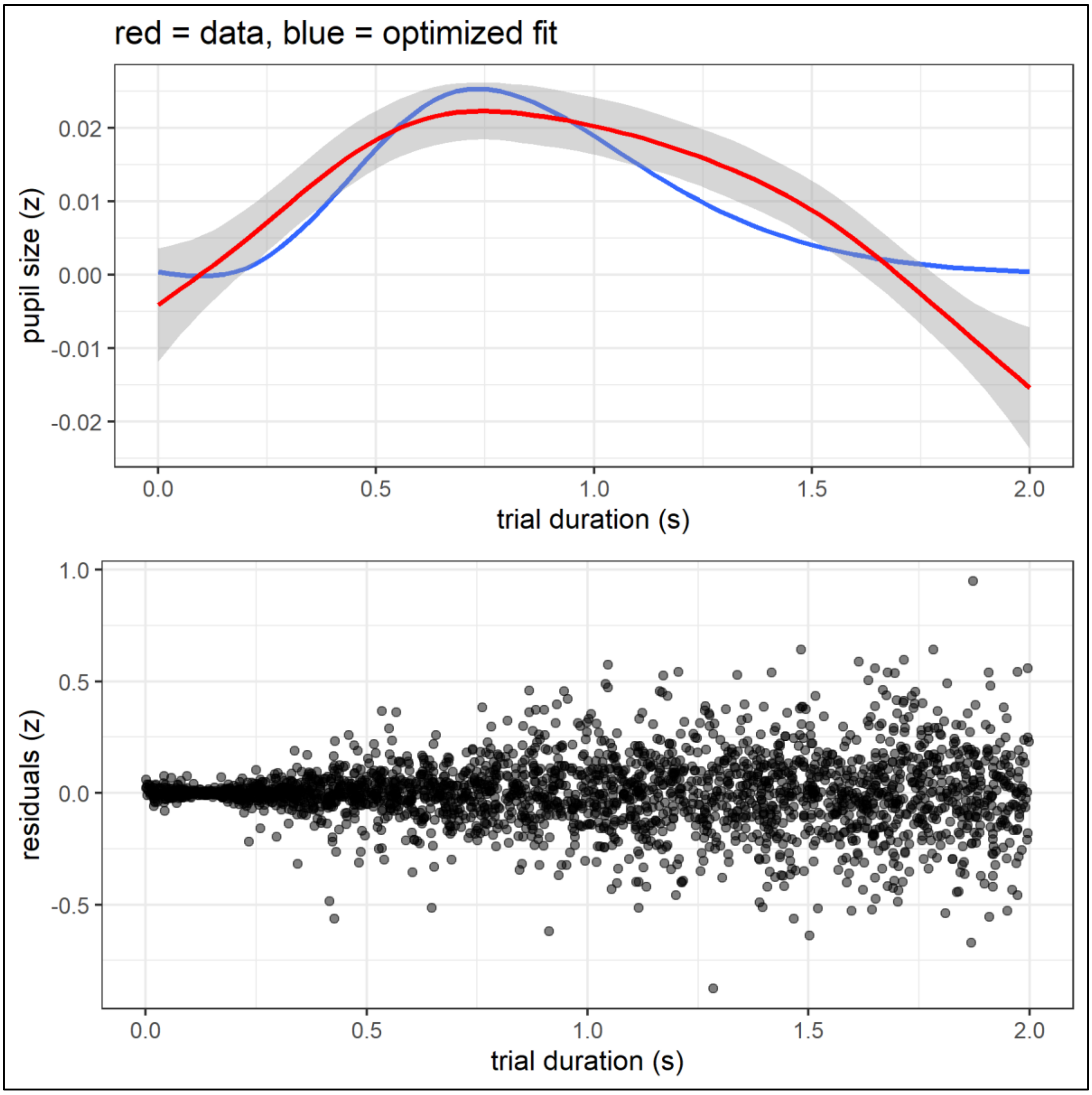

Figure S2. The canonical pupillary response function (CPF) was estimated in an independent data set of a passive, auditory oddball task in adolescents. Top: Red - Data of the pupillary response for stimuli. Blue – Optimized fit of a gamma function (shape = 6.65 [6.47, 6.82], rate = 7.68 [7.47, 7.89]) to replicate the pupillary response. This optimized fit is used as a CPF in the epoched data of the current study. The fit is $R^2 = 0.69$. Bottom: Residuals of the fit over time of a trial. Residuals appear randomly distributed with increasing variation over time. The R code documentation for the generation of the CPF can be found here: https://github.com/nicobast/oddball_LEAP/blob/master/analysis/data_preprocessing_oddball_leap.Rmd

Figure S3 – Sample trial of LTI-filtered pupillary response

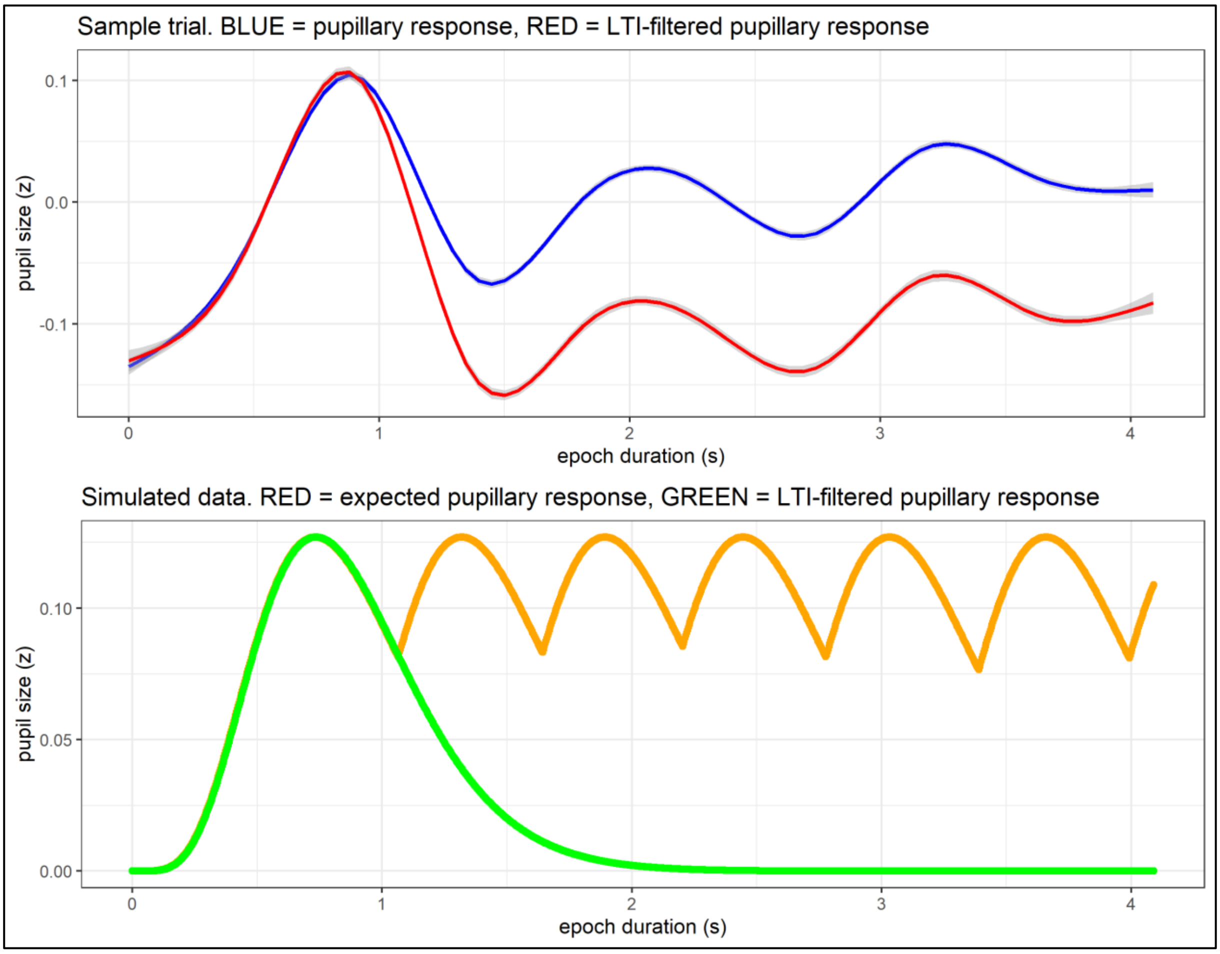

Figure S3. Sample trial of LTI-filtered pupillary response with the CPF (see Figure S2). Top: Randomly selected trial. Blue - Observed pupil size changes. Red - LTI-filtered pupillary response. Bottom: Visualization of simulated pupil size changes. Orange: Unscaled expected pupillary response to all trial within an epoch. Green: LTI-filtered pupillary response. The canonical pupillary response function (CPF) represents a gamma function. LTI = linear time-invariant.

Figure S4 – scatterplot of BPS and SEPR between timepoints

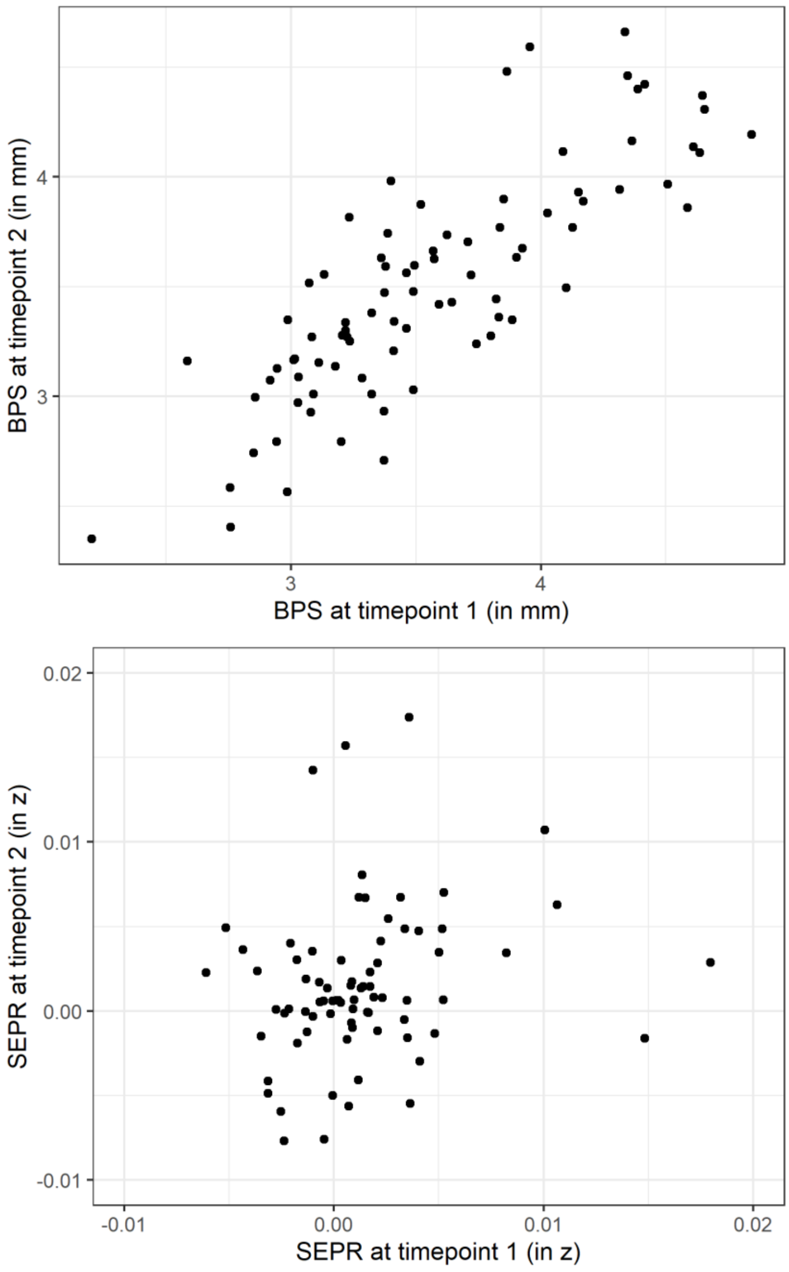

Figure S4. BPS = baseline pupil size, SEPR = stimulus evoked pupillary response.

Figure S5 – relative luminance across the task

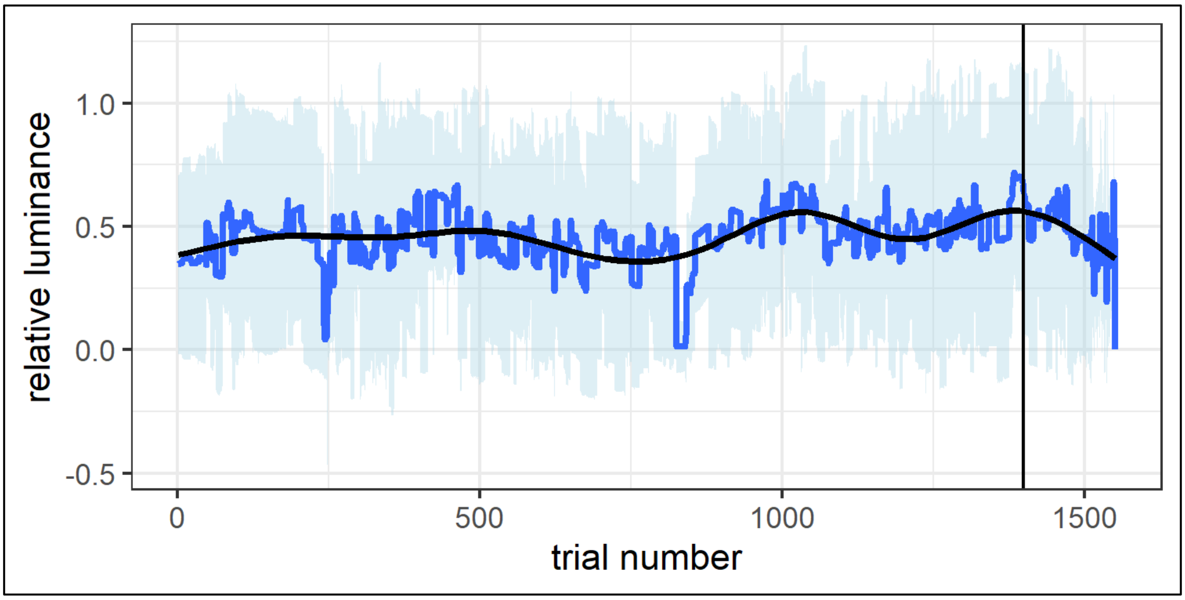

Figure S5. Relative luminance (0-1) of the video stimuli that is applied as a covariate in pupillary models to control for the effects of luminance on pupil size- The vertical line represents the end of the task (presentation of standard/oddball stimuli) while the video continued for some additional time. The graph shows that the tonic upregulation and downregulation of in baseline pupil size as an inverted u-shaped curve is unlikely to the induced by video luminance changes.

Figure S6 – data quality as missing data with task progression between groups

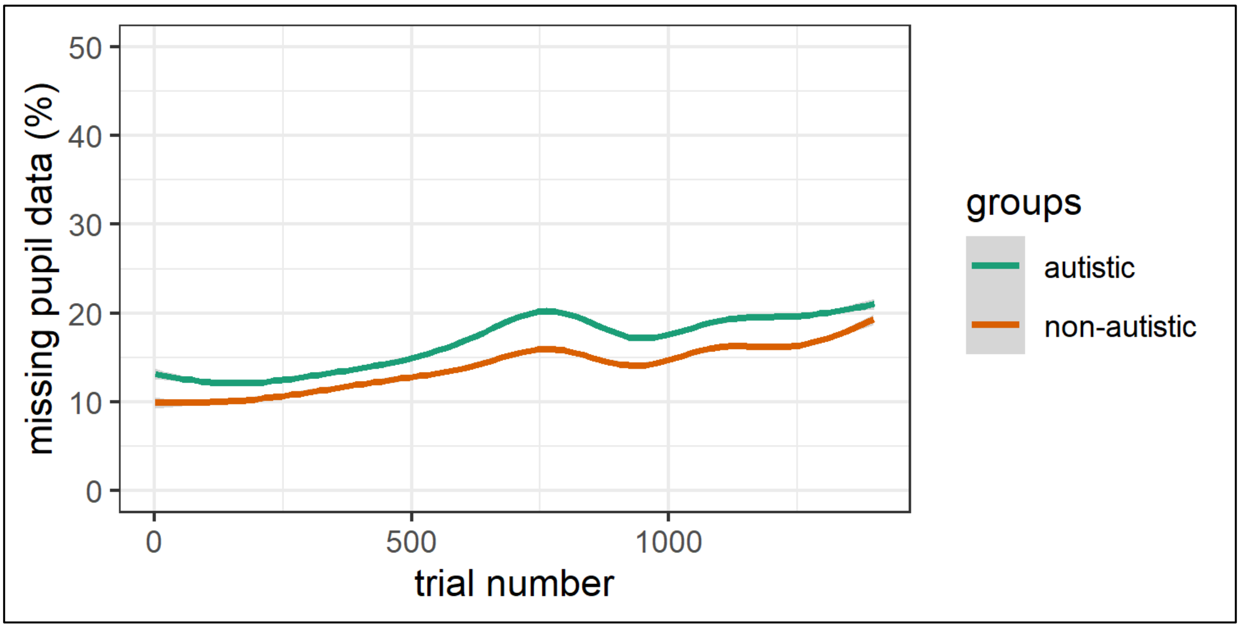

Figure S6. Missing pupil data (0-100%) with task progression as index of data quality between groups. The graph indicates a higher missing pupil data in autistic versus non-autistic individuals. In addition, missing pupil data increases with task progression. However, no systematic differences occur between groups with task progression supporting that dynamic changes of BPS and SEPR with task progression between groups are not driven by systematic changes of missing pupil data with task progression between groups. This variable is also considered in all statistical models.

Figure S7 – data quality as gaze center deviation with task progression between groups

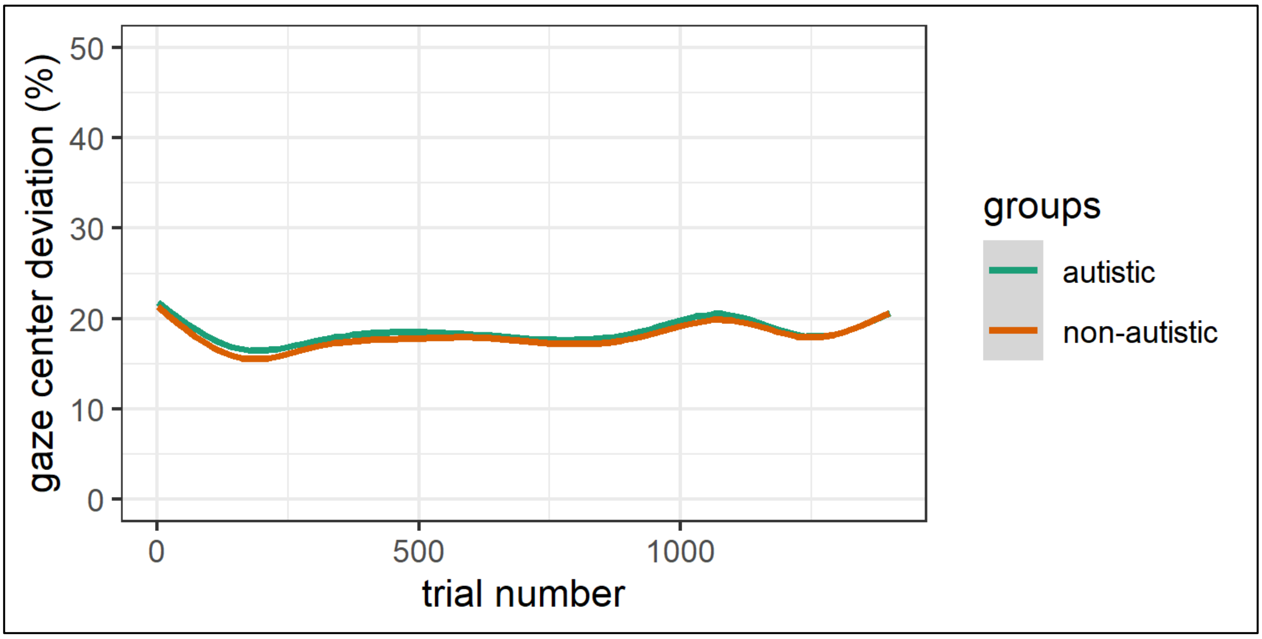

Figure S7. Gaze center deviation (0-100%) with task progression as index of data quality between groups. The graph indicates a slightly higher gaze center deviation in autistic versus non-autistic individuals. However, no systematic differences occur between groups with task progression supporting that dynamic changes of BPS and SEPR with task progression between groups are not driven by systematic changes of gaze center deviation with task progression between groups. This variable is also considered in all statistical models.

Figure S8 - histograms of per-participant estimates

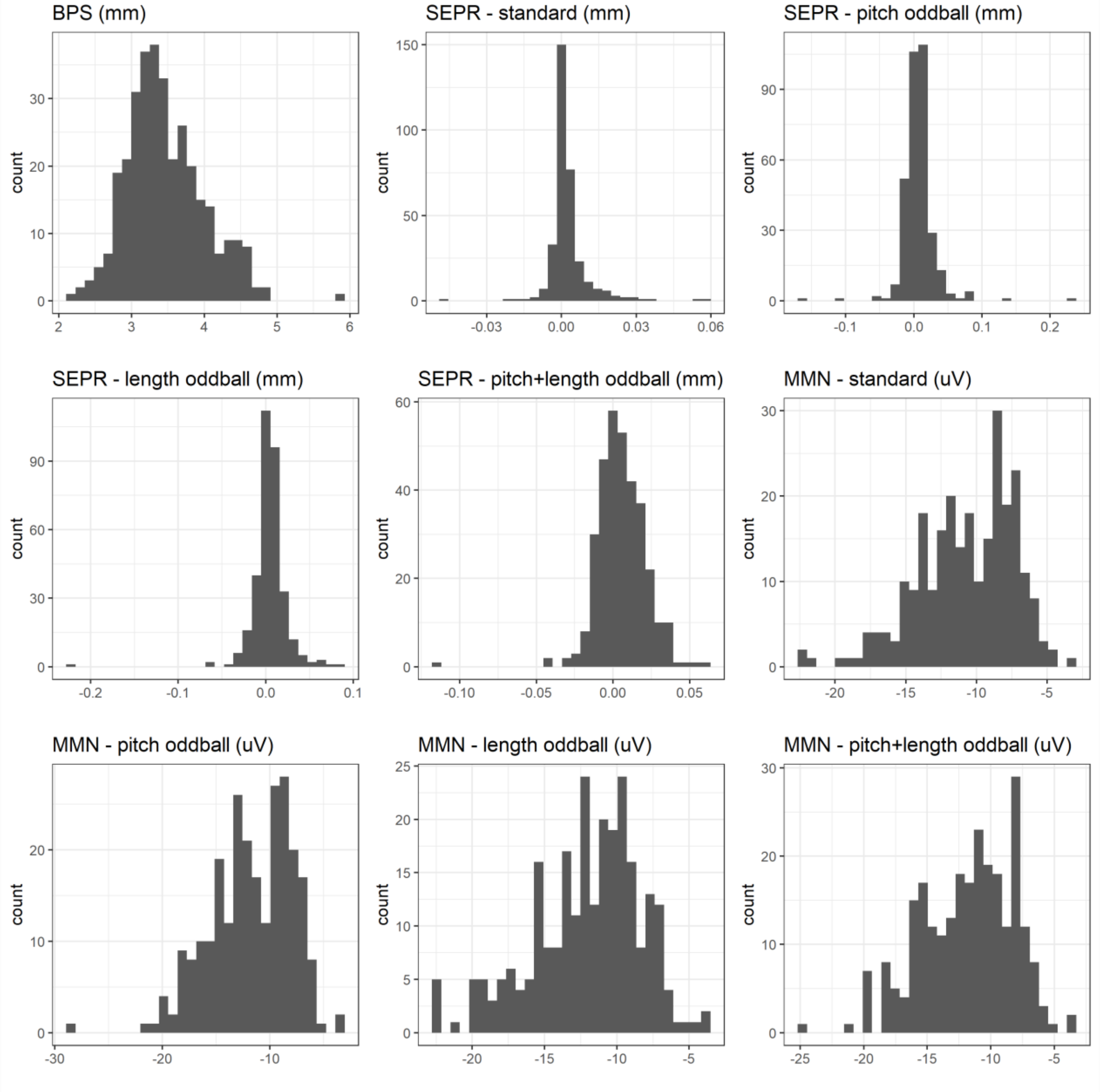

Figure S8. BPS = baseline pupil size, SEPR = stimulus evoked pupillary response, MMN = mismatch negativity.

Figure S9 - histograms of per-trial estimates

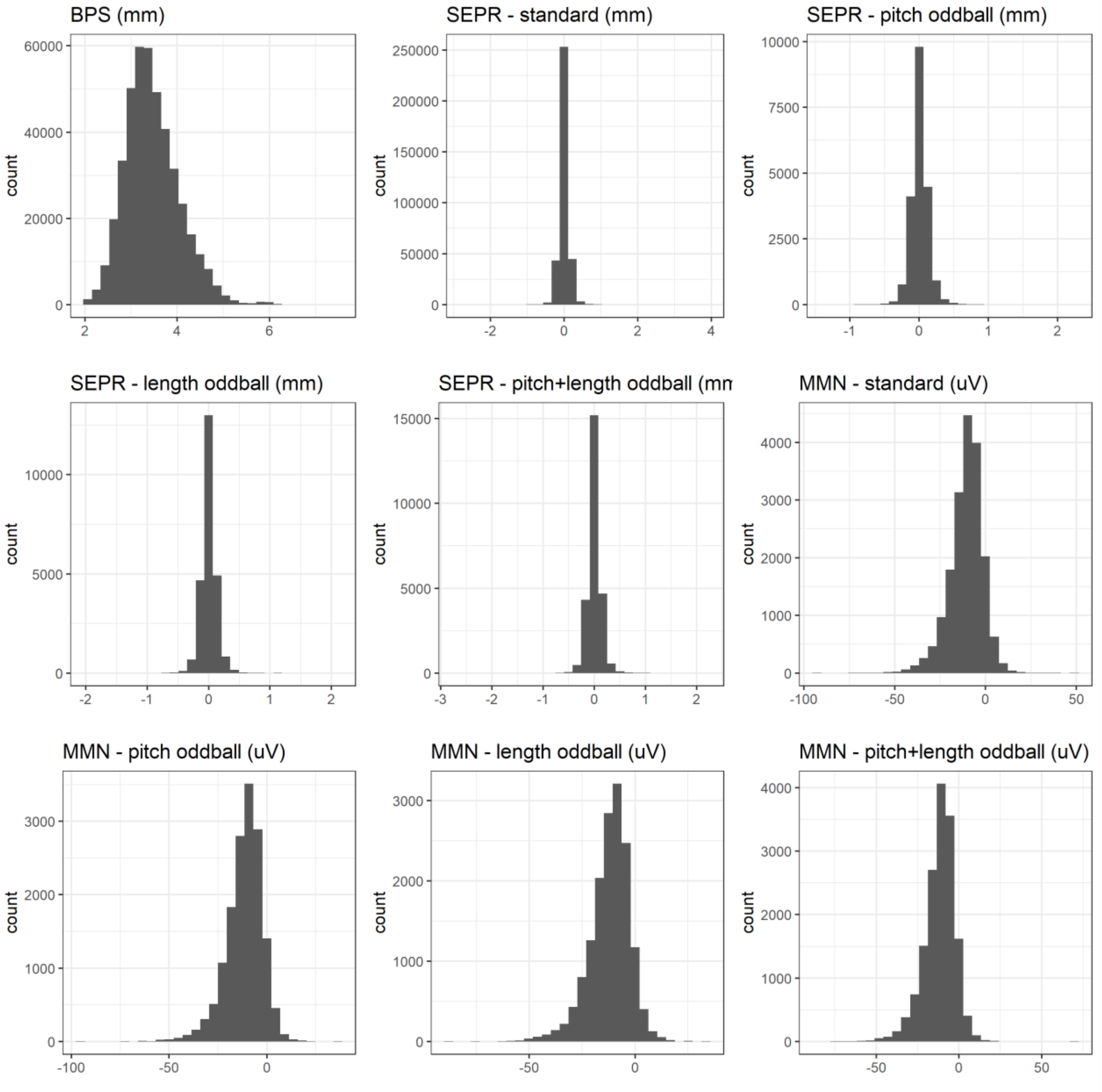

Figure S9. BPS = baseline pupil size, SEPR = stimulus evoked pupillary response, MMN = mismatch negativity.

Figure S10 - Bayesian posterior estimate MCMC chain convergence

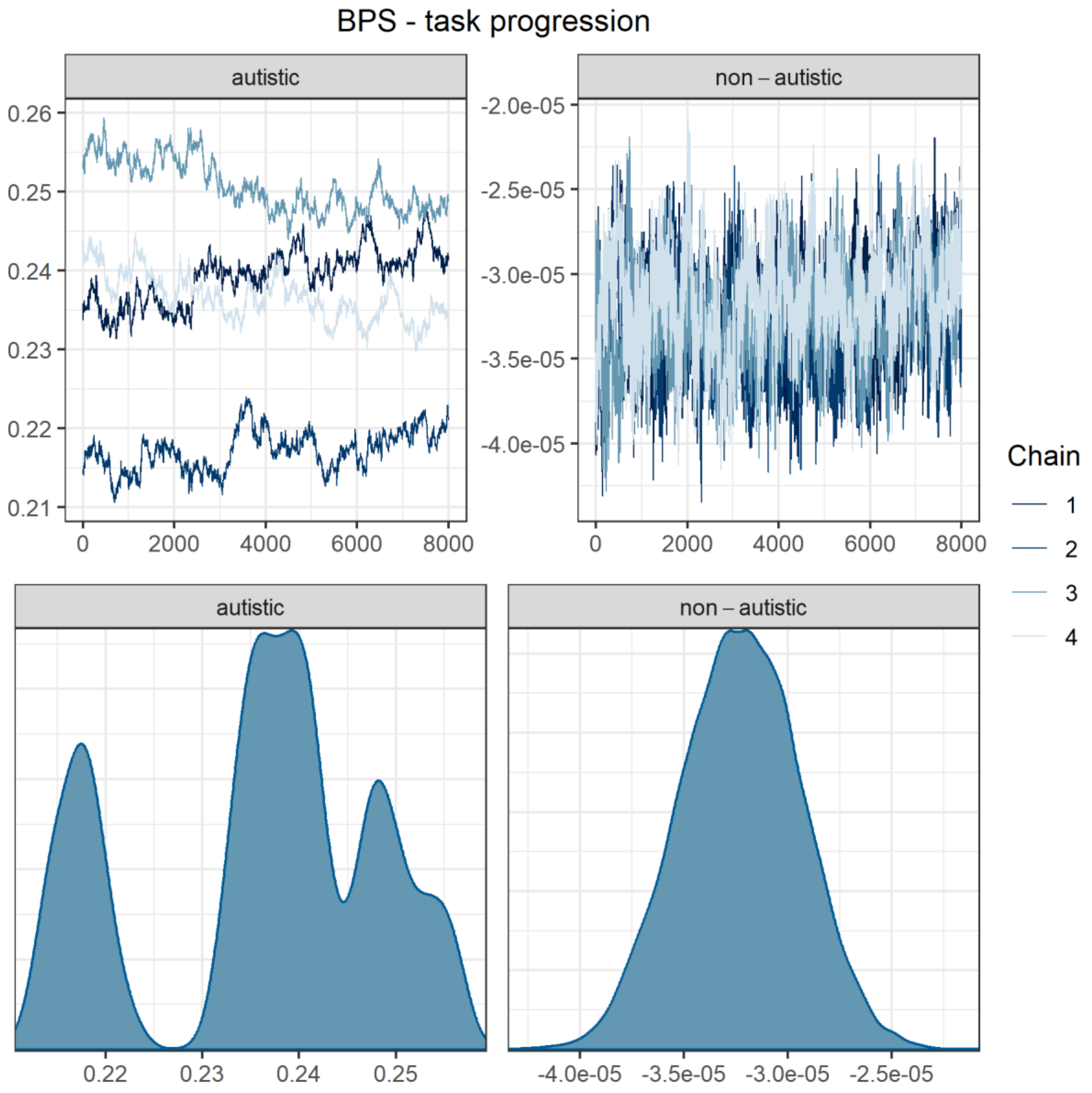

Figure S10. Top: Chain convergence of posterior estimates in autistic and non-autistic individuals. Bottom: Stationarity of posterior estimates in autistic and non-autistic individuals. MCMC = Markov Chain Monte Carlo, BPS = baseline pupil size.

Figure S11 - BPS association with age, IQ, biological sex

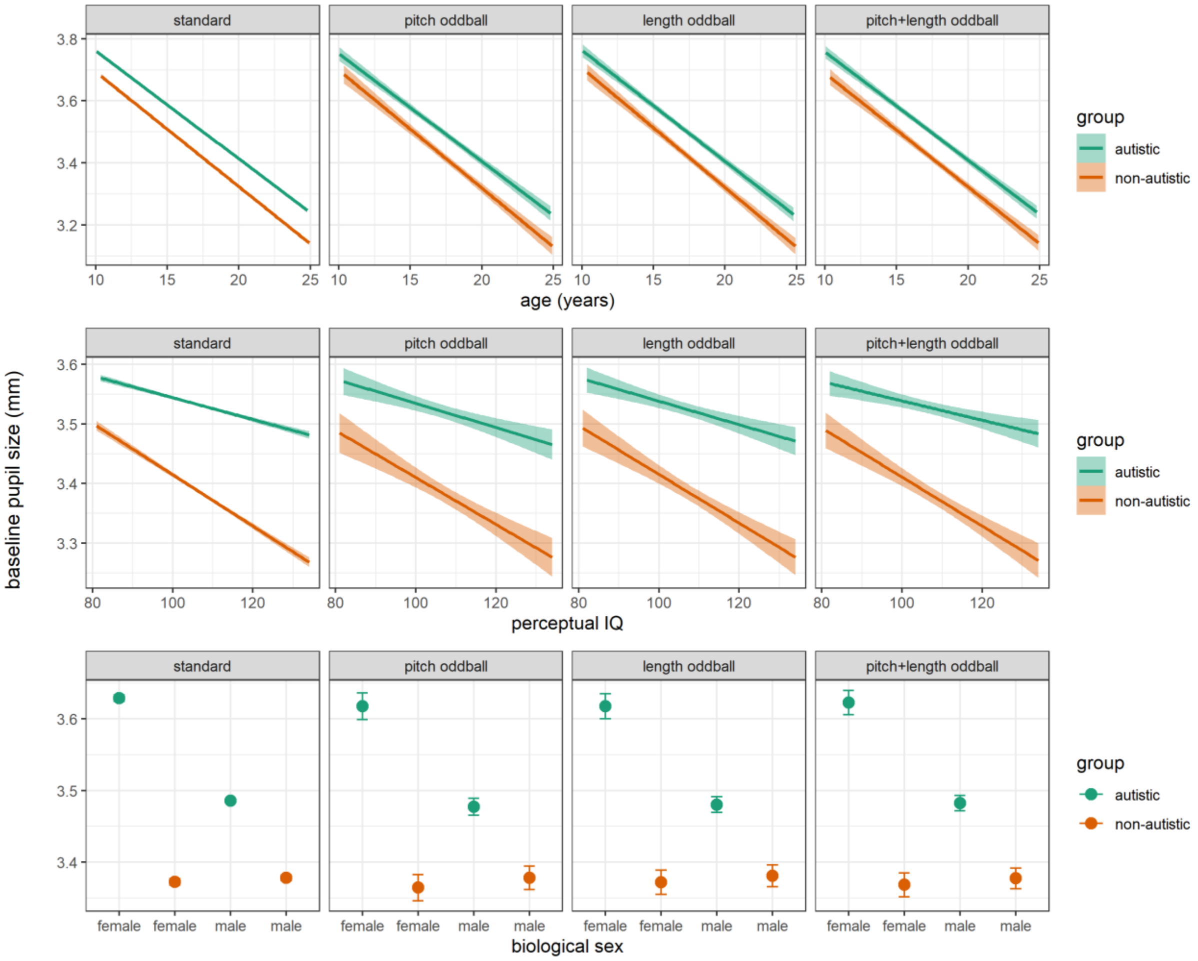

Figure S11. BPS = baseline pupil size.

Figure S12 - SEPR association with age, IQ, biological sex

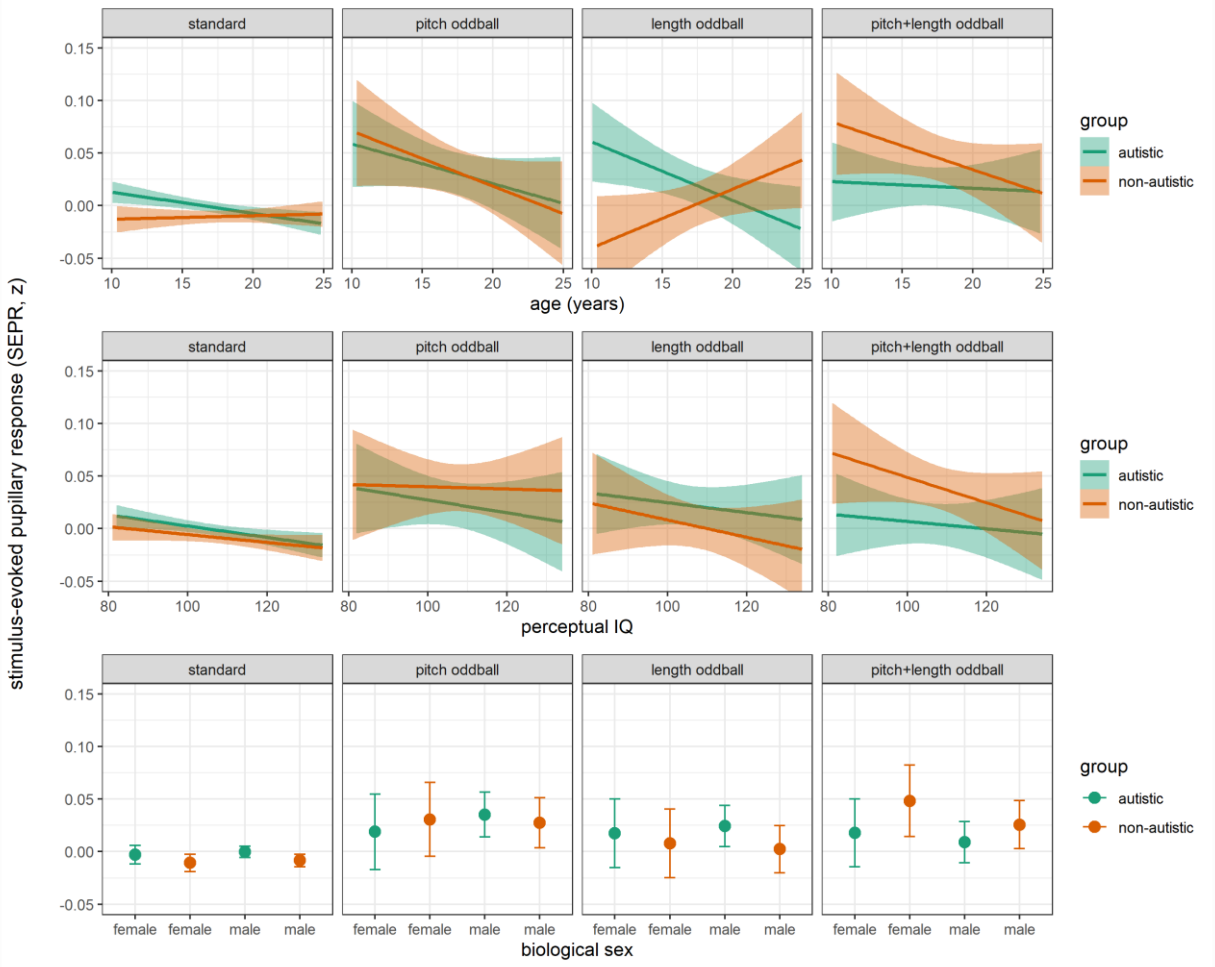

Figure S12. SEPR = stimulus evoked pupillary response.

Figure S13 - MMN-amp association with age, IQ, biological sex

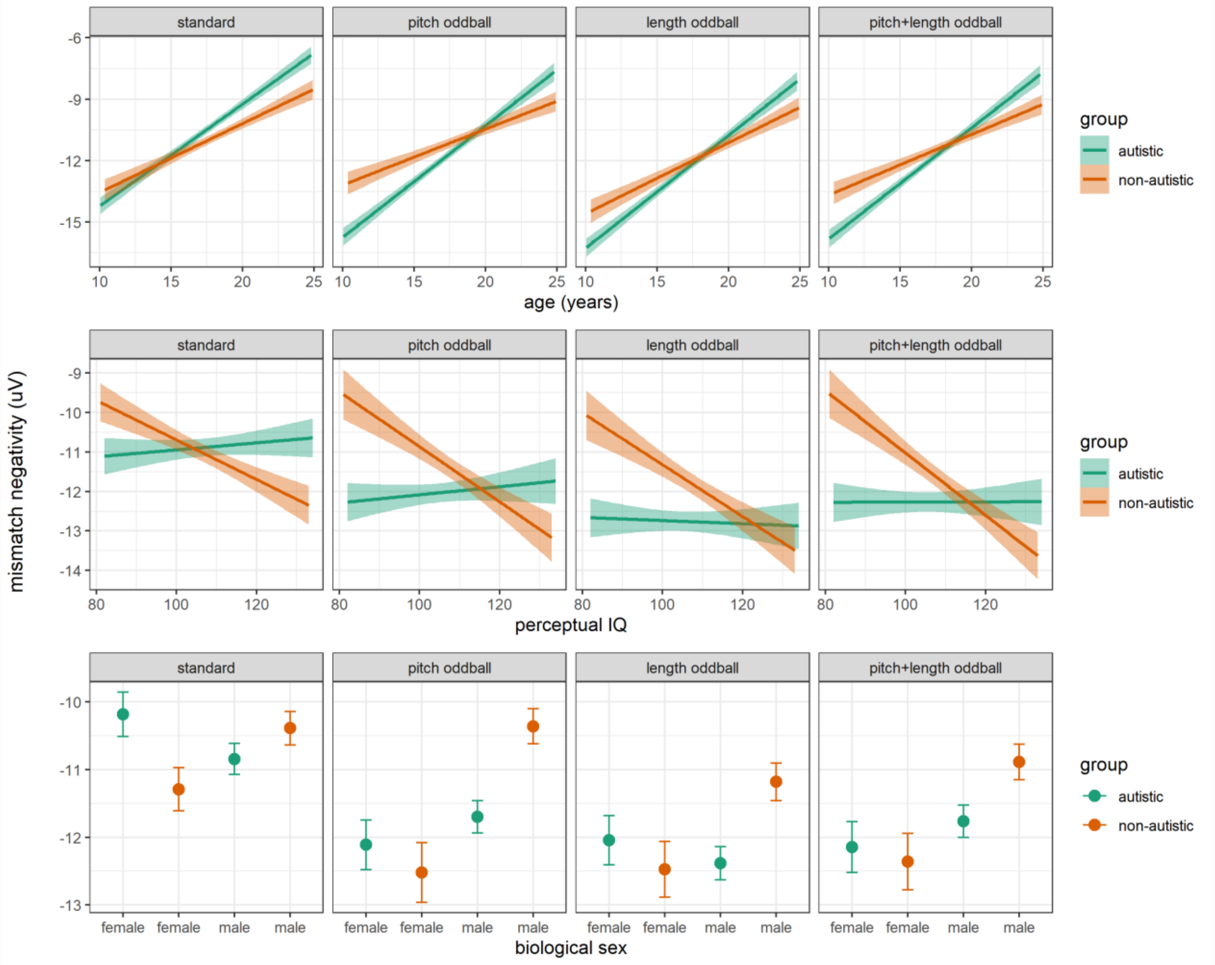

Figure S13. MMN-amp = mismatch-negativity-associated amplitude.

Figure S14 – data point distributions between stimulus conditions and with task progression

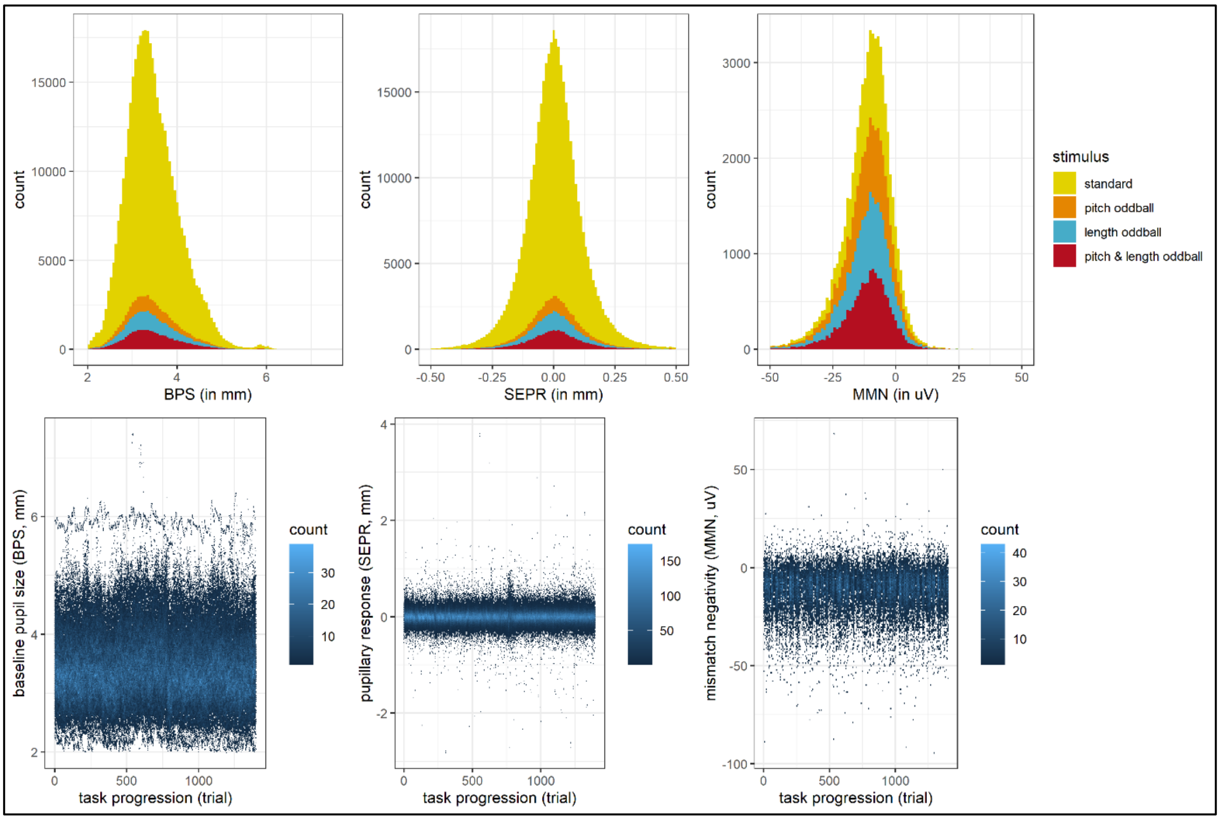

Figure S14. BPS = baseline pupil size, SEPR = stimulus evoked pupillary response, MMN = mismatch negativity.

Table S1 – descriptive statistics between sites

	KCL London (Tobii X-120)	CIMH Mannheim (Tobii TX-300)	group diff. (p)
n	184	54	-
gender (M/F)	126/58	40/14	0.536
timepoints (1/2/1+2)	41/80/63	27/4/23	<0.001
age (in years)	17.2/6.16 [6.24-30.98]	14.91/3.33 [6.57-20.94]	<0.001
IQ	99.69/22.59 [46-148]	105.14/15.42 [69-139]	0.048
perceptual IQ	100.01/22.03 [46-147]	106.5/16.73 [57-137]	0.022
verbal IQ	98.87/22.78 [45-160]	101.22/12.93 [73-130]	0.346
missing data per trial (%)	15.26/17.12 [0.46-99.98]	13.14/11.24 [0.41-42.97]	0.286
gaze center deviation (%)	17.91/2.4 [13.39-26.9]	18.96/2.15 [15.42-25.53]	0.003
BPS	3.44/0.54 [2.2-4.84]	3.56/0.52 [2.71-5.9]	0.096
SEPR - standard	0/0.01 [-0.05-0.06]	0/0.01 [-0.01-0.02]	0.304
SEPR - pitch	0.01/0.03 [-0.17-0.23]	0.01/0.01 [-0.02-0.05]	0.599
SEPR - length	0.01/0.02 [-0.22-0.09]	0/0.02 [-0.04-0.05]	0.055
SEPR - pitch + length	0/0.02 [-0.11-0.05]	0.01/0.01 [-0.02-0.06]	0.001
MMN - standard	-10.77/3.37 [-21.33--3.06]	-10.52/3.59 [-22.09--5.66]	0.627
MMN - pitch	-11.8/3.87 [-28.11--3.06]	-11.31/3.37 [-18.14--5.59]	0.334
MMN - length	-12.16/3.98 [-22.48--3.87]	-11.95/2.9 [-19.9--6.56]	0.656
MMN - pitch + length	-11.84/3.72 [-25.18--4]	-11.8/3.37 [-18.18--6.05]	0.93

Sample description between sites. BPS = baseline pupil size, SEPR = stimulus evoked pupillary response, MMN = mismatch negativity.

Table S2 - descriptive statistics on participant level

variable	n	mean	sd	min	max	skew	kurtosis
BPS	331	3.468	0.536	2.202	5.899	0.619	0.677
SEPR - standard	331	0.003	0.008	-0.047	0.059	1.870	16.518
SEPR - pitch oddball	331	0.007	0.025	-0.166	0.228	1.487	27.677
SEPR - length oddball	331	0.004	0.021	-0.220	0.088	-3.224	37.762
SEPR - pitch+length oddball	329	0.005	0.016	-0.114	0.061	-0.948	9.235
MMN - standard	257	-10.706	3.423	-22.095	-3.065	-0.639	0.210
MMN - pitch oddball	256	-11.672	3.751	-28.107	-3.055	-0.599	0.520
MMN - length oddball	256	-12.107	3.728	-22.484	-3.872	-0.583	0.049
MMN - pitch+length oddball	256	-11.833	3.626	-25.176	-4.002	-0.486	-0.102

Per-participant level: Descriptive statistics of baseline pupil size (BPS in mm), stimulus-evoked pupillary response (SEPR in mm), and mismatch negativity (MMN in μV).

Table S3 - descriptive statistics on trial level

variable	n	mean	sd	min	max	skew	kurtosis
BPS	427296	3.462	0.586	1.982	7.415	0.671	0.914
SEPR - standard	347627	0.002	0.128	-2.811	3.805	0.235	21.337
SEPR - pitch oddball	20595	0.007	0.127	-1.345	2.275	0.442	11.226
SEPR - length oddball	24512	0.005	0.128	-2.039	2.043	0.283	17.611
SEPR - pitch+length oddball	25489	0.006	0.132	-2.776	2.118	-0.273	20.083
MMN - standard	18227	-10.691	9.403	-91.488	50.221	-0.805	2.975
MMN - pitch oddball	15295	-11.521	9.502	-94.139	37.635	-1.014	3.253
MMN - length oddball	15404	-12.012	9.673	-88.872	32.803	-0.901	2.630
MMN - pitch+length oddball	15436	-11.678	9.537	-87.129	68.538	-0.828	3.210

Per-trial level: Descriptive statistics of baseline pupil size (BPS in mm), stimulus-evoked pupillary response (SEPR in mm), and mismatch negativity (MMN in µV).

Table S4 - group comparisons on per-participant estimates

	F	df1	df2	b	std. error	p
BPS - across stimuli	6.645	1	329	-0.287	0.111	0.01
SEPR - standards	2.456	1	329	-0.175	0.112	0.118
SEPR - pitch oddball	0.092	1	329	-0.034	0.112	0.761
SEPR - length oddball	0.073	1	329	-0.03	0.112	0.787
SEPR - pitch+length oddball	1.818	1	327	0.151	0.112	0.178
MMN - standards	0.673	1	255	0.104	0.127	0.413
MMN - pitch oddball	2.157	1	254	0.186	0.127	0.143
MMN - length oddball	2.645	1	254	0.206	0.127	0.105
MMN - pitch+length oddball	1.183	1	254	0.138	0.127	0.278

Per-participant level: Group comparisons of baseline pupil size (BPS), stimulus-evoked pupillary response (SEPR), and mismatch negativity (MMN).

Table S5 - covariate effects: BPS group difference

	Sum Sq	df	F	p
intercept	0.287	1	0.360	0.549
group	2.156	1	2.705	0.101
age	46.796	1	58.711	0.000
perceptual IQ	2.643	1	3.315	0.070
sex	1.158	1	1.453	0.229
sampling rate	1.272	1	1.595	0.207
gaze center deviation	6.620	1	8.306	0.004
data quality	0.003	1	0.004	0.951
residuals	257.449	323	NA	NA

Per participant: Full Linear model of baseline pupil size (BPS) with all potential covariates.

	Sum Sq	df	F	p
intercept	1.022	1	1.271	0.260
group	2.456	1	3.053	0.082
age	43.779	1	54.437	0.000
gaze center deviation	10.049	1	12.496	0.000
residuals	262.974	327	NA	NA

Per participant: Reduced Linear model of baseline pupil size (BPS) with significant covariates.

Table S6 - correlation table of BPS, SEPR, MMN-amp, NG

correlation	coefficient (r)	p	p adjusted
SEPR (standard) and BPS	0.164	0.003	0.036
SEPR (pitch oddball) and BPS	0.043	0.436	1.000
SEPR (length oddball) and BPS	0.167	0.002	0.024
SEPR (pitch+length oddball) and BPS	0.060	0.279	1.000
SEPR (standard) and MMN (standard)	-0.200	0.001	0.012
SEPR (pitch oddball) and MMN (pitch oddball)	-0.143	0.022	0.264
SEPR (length oddball) and MMN (length oddball)	-0.121	0.054	0.648
SEPR (pitch+length oddball) and MMN (pitch+length oddball)	-0.027	0.669	1.000
BPS and MMN (standard)	-0.177	0.004	0.048
BPS and MMN (pitch oddball)	-0.195	0.002	0.024
BPS and MMN (length oddball)	-0.214	0.001	0.012
BPS and MMN (pitch+length oddball)	-0.213	0.001	0.012

Correlations of baseline pupil size (BPS), stimulus-evoked pupillary response (SEPR), and mismatch negativity (MMN) for different stimuli.

Table S7 – SEPR regression model with BPS as predictor between groups

	Sum Sq	df	F	p
intercept	0.330	1	0.342	0.559
BPS	12.028	1	12.468	0.000
group	1.577	1	1.634	0.202
BPS x group	4.335	1	4.494	0.035
residuals	315.466	327	NA	NA

Per participant: Regression model of stimulus-evoked response (SEPR) to standards with baseline pupil size (BPS) as predictor between groups.

Table S8 – SEPR regression model with MMN as predictor between groups

	Sum Sq	df	F	p
intercept	0.309	1	0.397	0.529
MMN	6.840	1	8.784	0.003
group	4.423	1	5.680	0.018
MMN x group	0.386	1	0.496	0.482
residuals	196.218	252	NA	NA

Per participant: Regression model of stimulus-evoked response (SEPR) to standards with mismatch negativity (MMN) to standards as predictor between groups.

Table S9 - polynomial fit comparison for BPS, SEPR; MMN-amp

	number of parameters	AIC	BIC	log likelihood	deviance	Chi-squared	df	p-value
linear fit	4	475043.0	475086.9	-237517.5	475035.0	NA	NA	NA
quadratic fit	5	467752.9	467807.7	-233871.4	467742.9	7292.1632	1	0
cubic fit	6	467614.5	467680.3	-233801.3	467602.5	140.3606	1	0

	number of parameters	AIC	BIC	log likelihood	deviance	Chi-squared	df	p-value
linear fit	4	1186583	1186627	-593287.5	1186575	NA	NA	NA
quadratic fit	5	1186584	1186639	-593287.1	1186574	0.7436042	1	0.389
cubic fit	6	1186577	1186643	-593282.7	1186565	8.7644318	1	0.003

	number of parameters	AIC	BIC	log likelihood	deviance	Chi-squared	df	p-value
linear fit	4	175331.9	175368.2	-87661.94	175323.9	NA	NA	NA
quadratic fit	5	175328.7	175374.1	-87659.36	175318.7	5.148342	1	0.023
cubic fit	6	175324.7	175379.2	-87656.37	175312.7	5.976667	1	0.014

Model comparison on the linearity of trial number on mismatch negativity (MMN).

Table S10 - BPS linear mixed model - task progression

	Sum Sq	Mean Sq	df1	df2	F	p adjusted
stimulus (S)	1.778	0.593	3	425391.030	3.629	0.012
group	0.693	0.693	1	311.290	4.246	0.040
task progression (TP)	360.456	120.152	3	425392.199	735.892	0.000
video luminance	100.995	100.995	1	425391.183	618.563	0.000
age	10.808	10.808	1	311.054	66.192	0.000
perceptual IQ	0.504	0.504	1	311.004	3.089	0.080
sex	0.330	0.330	1	311.007	2.024	0.156
sampling rate	0.977	0.977	1	311.032	5.983	0.015
gaze center deviation	538.530	538.530	1	425427.173	3298.322	0.000
data quality	1170.123	1170.123	1	425509.275	7166.625	0.000
S x group	1.765	0.588	3	425391.029	3.603	0.013
S x TP	25.589	2.843	9	425391.035	17.414	0.000
group x TP	23.161	7.720	3	425391.758	47.285	0.000
S x group x TP	1.824	0.203	9	425391.025	1.242	0.264

Linear mixed model: baseline pupil size (BPS) - task progression.

covariate	estimate	lower bound (2.5%)	upper bound (97.5%)
video luminance	-0.02	-0.03	-0.01
age	-0.37	-0.46	-0.28
sampling rate (300Hz vs. 120Hz)	0.23	0.03	0.43
gaze center deviation	0.04	0.02	0.06
data quality / missing data	0.09	0.08	0.10

Covariate effects on baseline pupil size (BPS).

Supplementary Note 1 - effect of medication on group differences

A higher proportion of autistic versus non-autistic individuals received medication (ASD: 46.4%, non-ASD: 20.4%, $Chi^2 = 15.88$, $p < .001$). 11 autistic and 3 non-autistic participants received more than one medication. Medication was primarily related to comorbid symptoms in those receiving medication (melatonin: 23%, methylphenidate: 22%, selective serotonin reuptake inhibitor: 15%, antiepileptics: 11%, risperidone: 7%, atomoxetine: 6%, other: 16%). We calculated a variable whether participants received any medication (yes versus no), which was included as a covariate into models that reported group differences.

On a per participant level, the inclusion of medication did not influence baseline pupil size (BPS) and did not alter the group differences (BPS: $F(1, 328) = 6.63$, $p = .010$). For the dynamic BPS model, medication also had no significant effect on the cubic fit of BPS ($F(1, 317) < 1$) and did not alter the dynamic group difference (group x task progression: F(3, 426980 = 42.27, $p < .001$).

For the dynamic SEPR and MMN model, medication did not have a significant effect on SEPR or MMN ($F < 1$).
